# Supplementary material for: Ancestral L-amino acid oxidases for deracemization and stereoinversion of amino acids
Source: Commun Chem. 2020 Dec 4;3:181. doi: 10.1038/s42004-020-00432-8 (PMC9814856; doi:10.1038/s42004-020-00432-8)
Supplement: Supplementary file 2 — Supplementary Information [file 42004_2020_432_MOESM2_ESM.pdf]

Supplementary information

Ancestral L-amino acid oxidases for deracemization and stereoinversion of amino acids

Shogo Nakano[#, 1, *], Kohei Kozuka[#, 1], Yuki Minamino[1], Hiroka Karasuda[1], Fumihito Hasebe[1] and Sohei Ito[1]

[1]Graduate School of Integrated Pharmaceutical and Nutritional Sciences, University of Shizuoka, 52-1 Yada, Suruga-ku, Shizuoka 422-8526, Japan

[*]Correspondence to Shogo Nakano (snakano@u-shizuoka-ken.ac.jp)

http://orcid.org/0000-0002-6614-7158 (Orcid link)

[#]These authors contributed equally to this work

Contents

・**Supplementary Figures** **S3-S17**

Multiple sequence alignment of designed AncLAAOs and AncAROD S3

Phylogenetic analysis of AncLAAOs, AncAROD and their homologs S4

Enzymatic properties of AncLAAO-N4 S5

Chiral HPLC chromatograms of D,L-**1a** and **1b** by AncLAAO-N4 S6

Chiral HPLC chromatograms of compounds from D,L-**1c** to **1e** by AncLAAO-N4 S7

Chiral HPLC chromatograms for stereoinversion of L-**1f**-**1h** by AncLAAO-N4 S8

Molecular weight analysis of AncLAAO-N5 by gel-filtration chromatography S9

Structural comparison between AncLAAO-N5 and LAAO from *C. rhodostoma* S10

Schematic view to screen AncLAAO-N4 variants which can oxidize L-Val efficiently S11

Enzyme kinetics plots of AncLAAO-N4 and their variants S12

Chiral HPLC chromatograms of D,L-Val by N4-D249V/Q536L/Y568F variant S13

HRMS analysis for products of deracemization and stereoinversion reaction S14-S18

・**Supplementary Table contents** **S19-S24**

Sequence identity between the AncLAAOs S19

Summary for purification of the AncLAAOs from 1L cultivation S19

Relative activity of AncLAAO-N4 and AncLAAO-N5 toward 20 L-amino acids S20

LC condition and retention time for amino acid derivatives S21

Top 10 of structures which have structural similarity to AncLAAO-N5 by Dali search S22

Primer list to prepare site-directed variants of AncLAAO-N4 S23

Enzyme kinetic parameters of AncLAAO-N4 and their variants to six L-amino acids S24

・**Supplementary References** **S25**

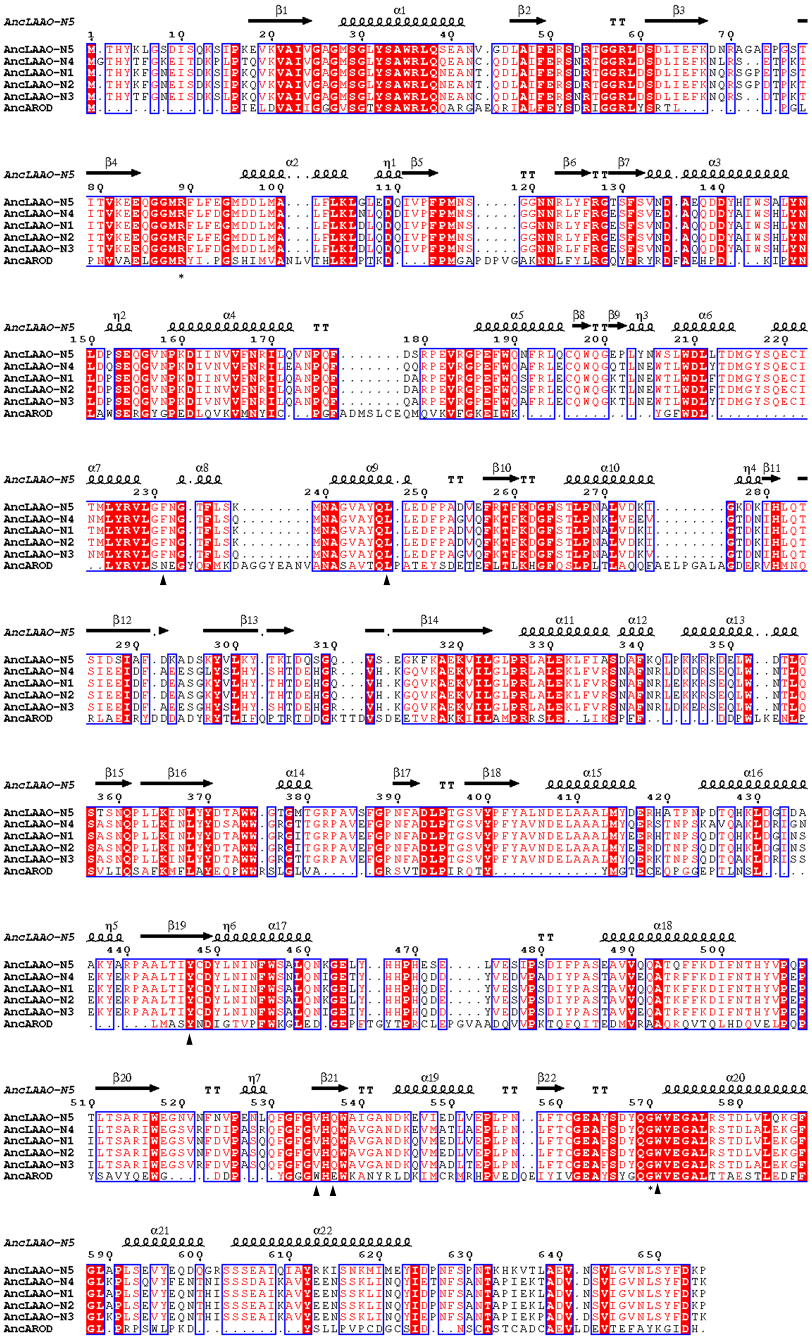

Supplementary Figure 1. Multiple sequence alignment of designed AncLAAOs (AncLAAO-N1, N2, N3, N4 and N5) and AncAROD. Abbreciation for AncAROD was L-arginine oxidase designed by ancestral sequence reconstruction method. The residues which form hydrogen bonds with substrate were shown as asterisk. The residues forming hydrophobic interactions were represented as arrow head. Alignment was performed using MAFFT (*1*), and the figure was generated by ESPript (*3*).

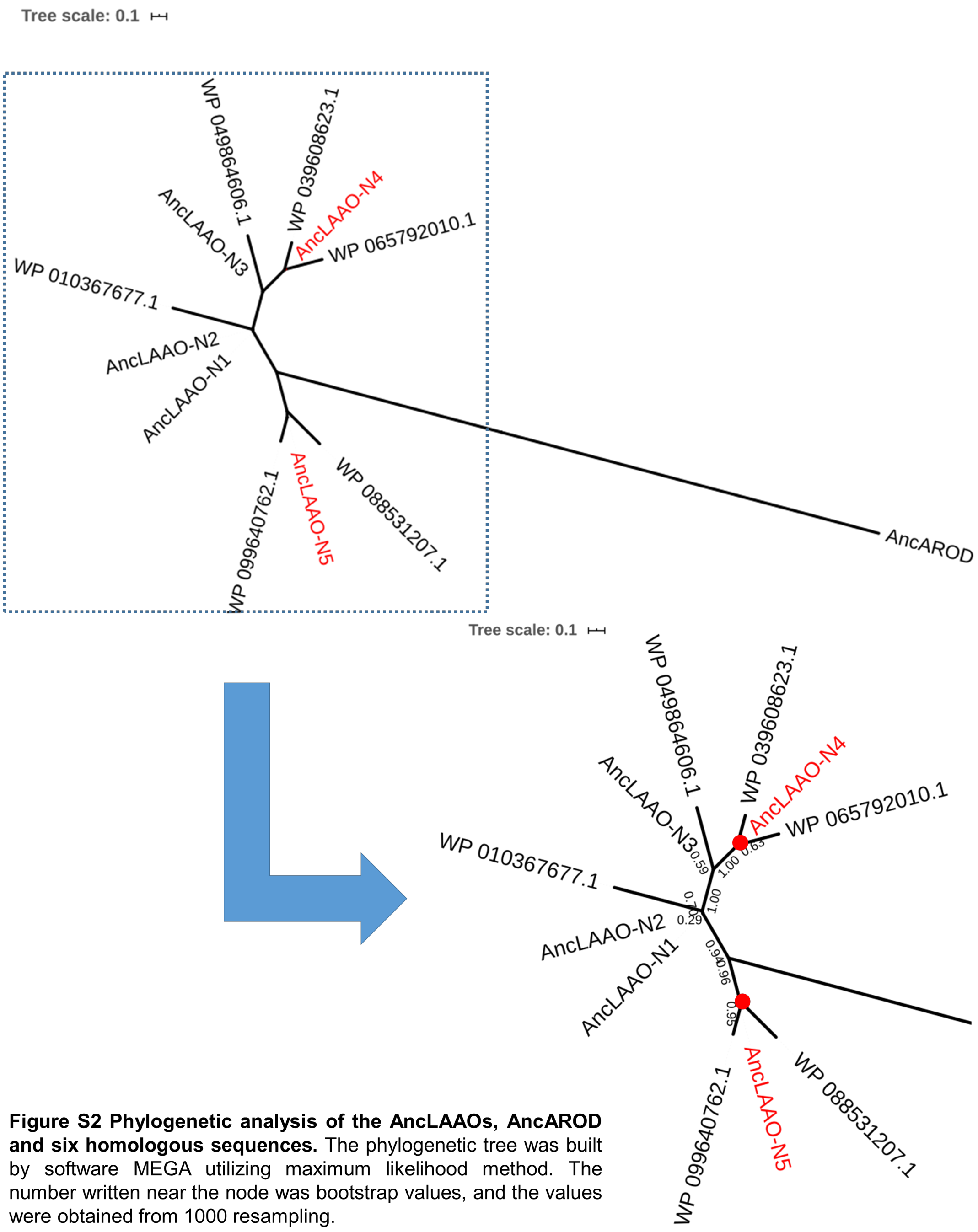

Figure S2 Phylogenetic analysis of the AncLAAOs, AncAROD and six homologous sequences. The phylogenetic tree was built by software MEGA utilizing maximum likelihood method. The number written near the node was bootstrap values, and the values were obtained from 1000 resampling.

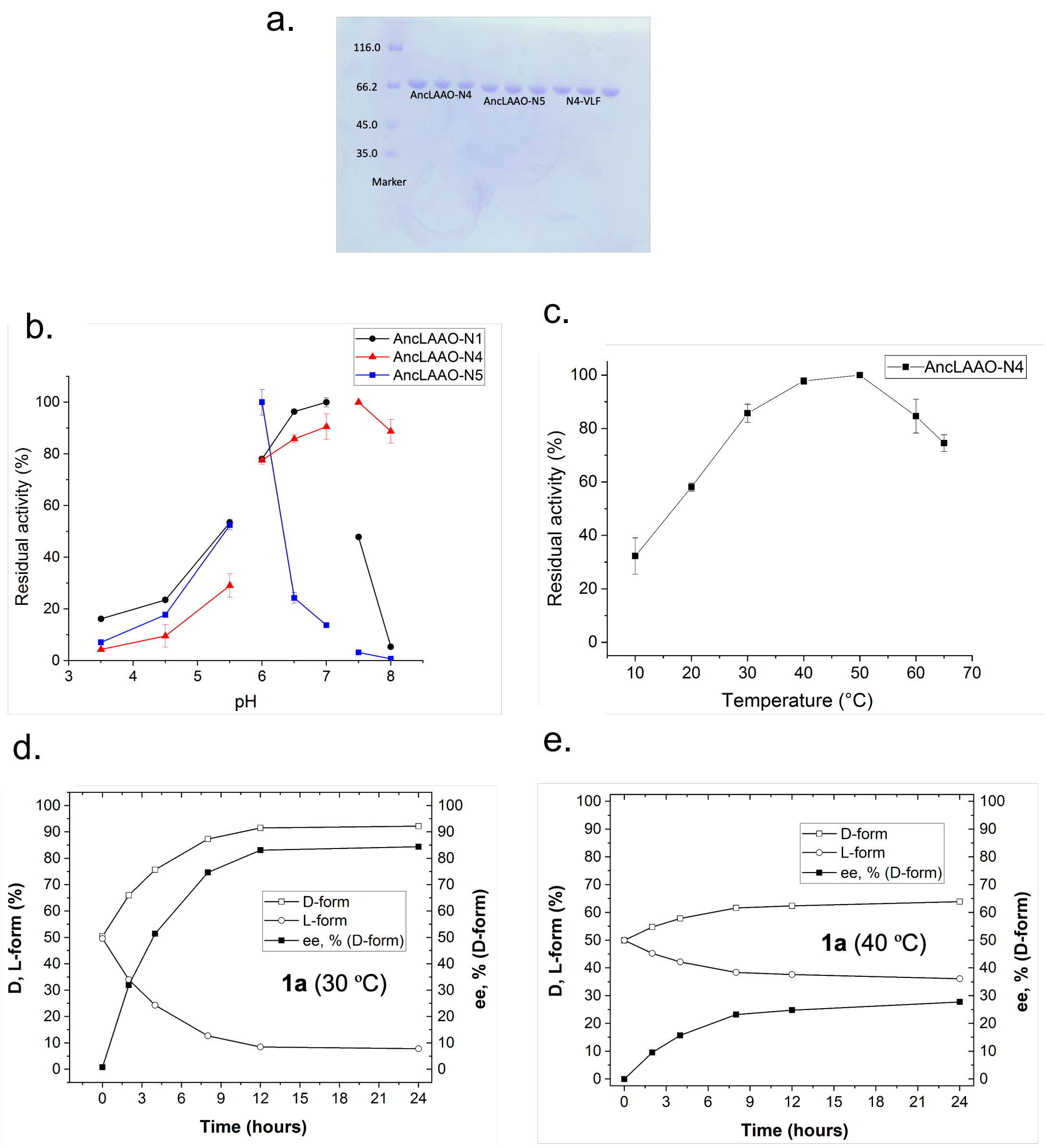

Supplementary Figure 3. SDS-PAGE for AncLAAO-N4, N5 and N4-VLF (a). Activity changes of AncLAAO-N1, N4 and N5 associated with pH change (b). Residual activity was calculated by regarding the highest activity of each sample as 100%. **Activity changes of AncLAAO-N4 associated with temperature change (c).** The activity at 50 ºC was set to 100%. Activity measurement was performed utilizing the identical assay buffer to the measurement of thermal stability. **Time course of D-1a production from racemates by deracemization reaction with AncLAAO-N4 at different temperature (d, e).** The reaction was performed under condition of 30 (d) and 40 ºC (e), respectively.

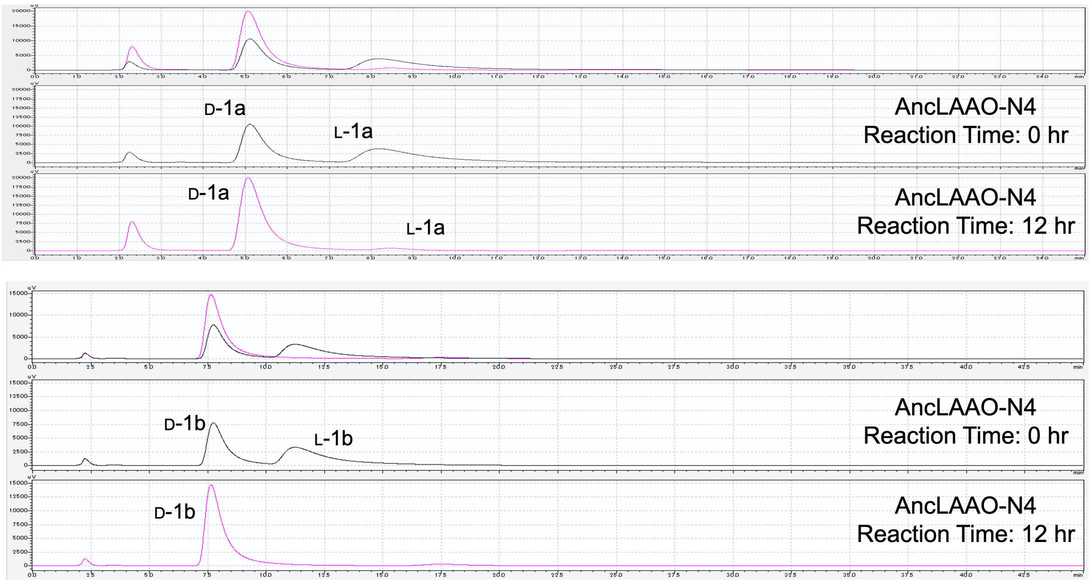

Supplementary Figure 4. Chiral HPLC chromatograms before (black line) and after deracemization (magenta) of racemic compounds (D,L-1a and 1b) by AncLAAO-N4 . The retention time and detailed condition about HPLC chromatograms were written in Material & method section and Table S6. The reactions were performed at preparative scale: Total 166 mg (D,L-**1a**) and 210 mg (D,L-**1b**) of substrates were applied to the deracemization. In any conditions, total 3 mg of purified AncLAAO-N4 were utilized in the reaction. Chemical assignment of the products (D-**1a** and **1b**) was performed by HRMS analysis (Supplementary Figure 12).

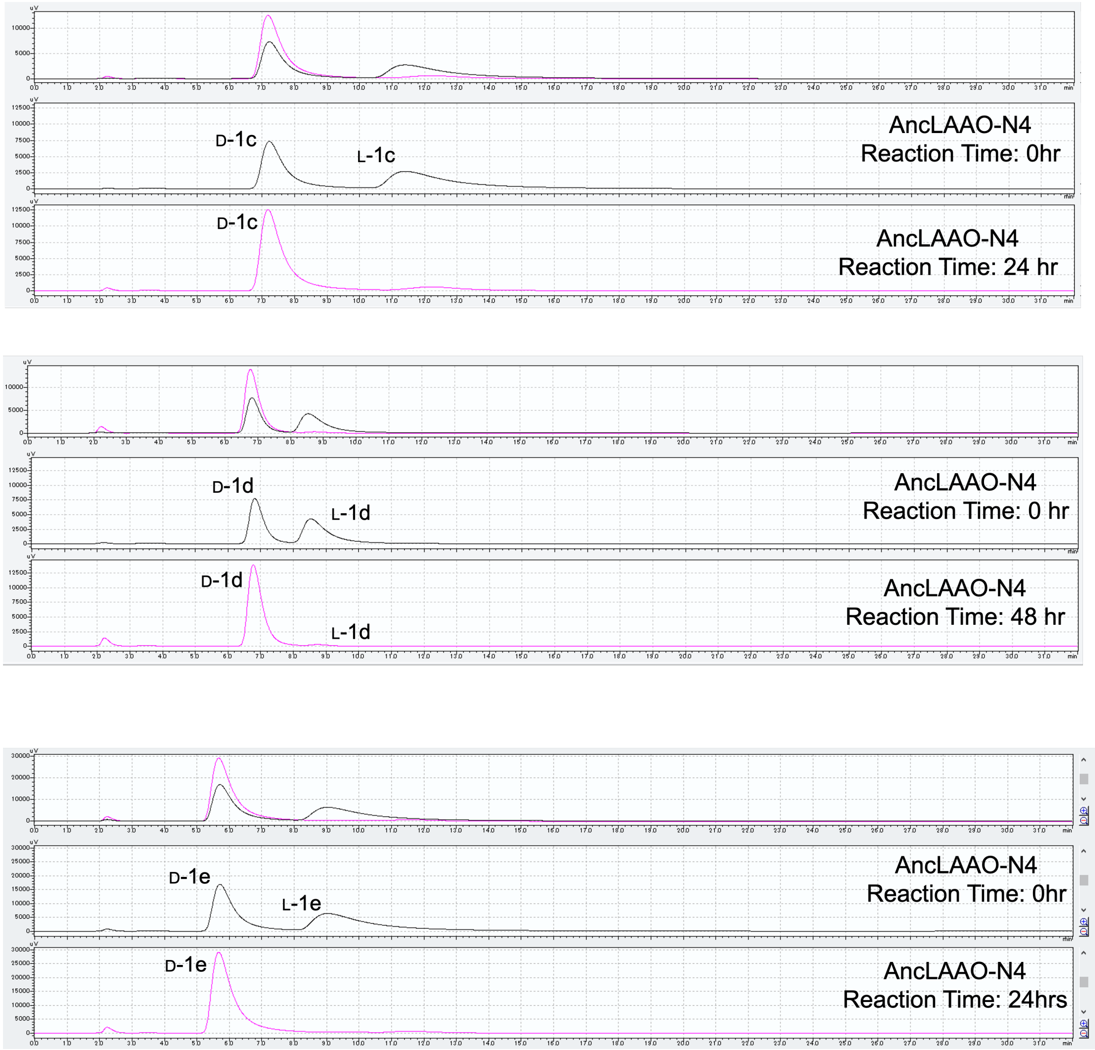

Supplementary Figure 5. Chiral HPLC chromatograms of compounds before (black line) and after deracemization (magenta) of phenylalanine derivatives by AncLAAO-N4. The retention time and detailed condition about HPLC chromatograms were written in Material & method section and Supplementary Table 4. The reactions were performed at preparative scale: Total 195 mg (D,L-**1c**), 180 mg (D,L-**1d**) and 183 mg (D,L-**1e**) of substrates were applied to the deracemization. In any conditions, total 2 mg of purified AncLAAOs were utilized in the reaction. Chemical assignment of the products (D-**1c**-**1e**) was performed by HRMS analysis (Supplementary Figure 12).

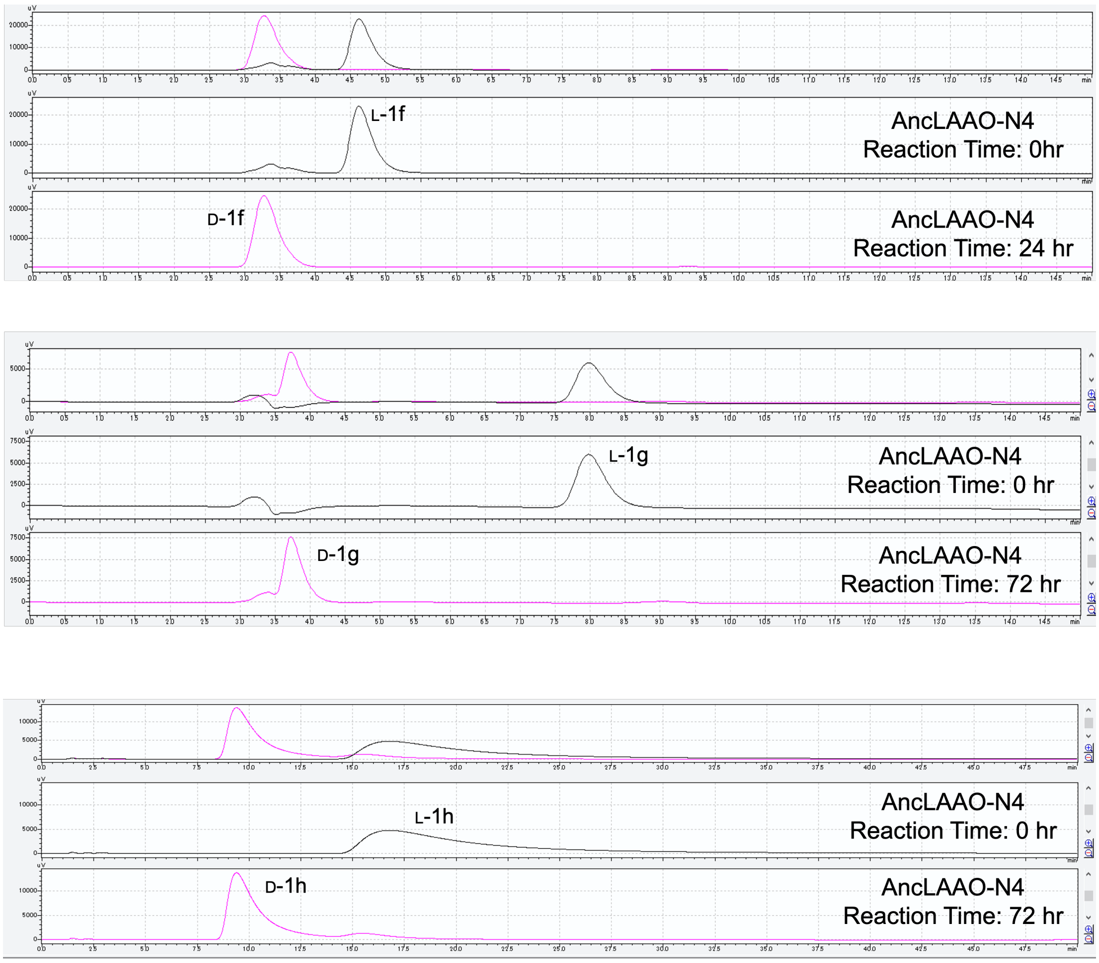

Supplementary Figure 6. Chiral HPLC chromatograms of compounds before (black line) and after stereoinversion (magenta) of amino acids by AncLAAO-N4. The retention time and detailed condition about HPLC chromatograms were written in Material & method section and Supplementary Table 4. The reactions were performed at preparative scale: Total 146 mg (L-**1f**), 147 mg (L-**1g**) and 204 mg (L-**1h**) of substrates were applied to the stereoinversion. In any conditions, total 2 mg of purified AncLAAOs were utilized in the reaction. For the calculation of conversion rate of **1f**, **1g, and 1h**, the peak area of D-isoforms was subtracted from the overlapped peak area confirmed in L-isoforms. Chemical assignment of the products (D-**1f**, **1g, and 1h**) was performed by HRMS analysis (Supplementary Figure 12).

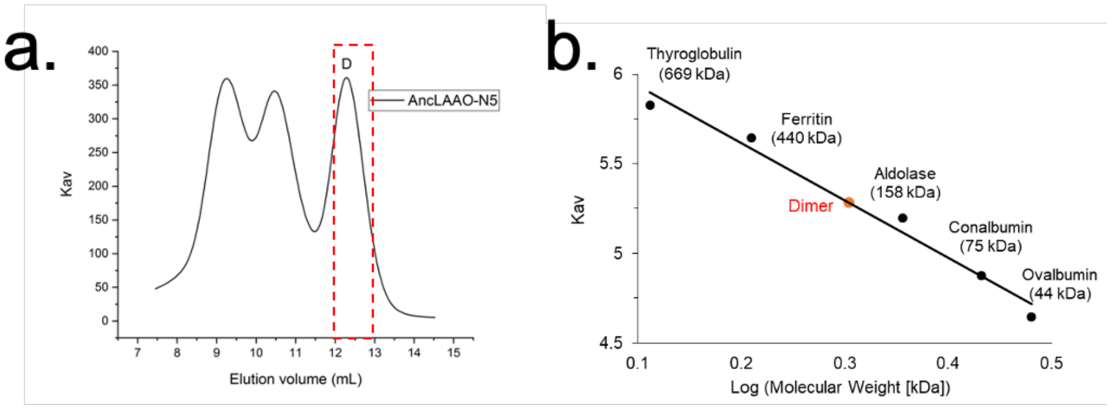

Supplementary Figure 7 Characterization of oligomeric state of AncLAAO-N5 which was utilized in the crystallization by SEC analysis. The fractions which were enclosed as red dotted square were collected and applied to the crystallization (a). From the calibration curve, AncLAAO-N5 in the fraction had dimer form (b).

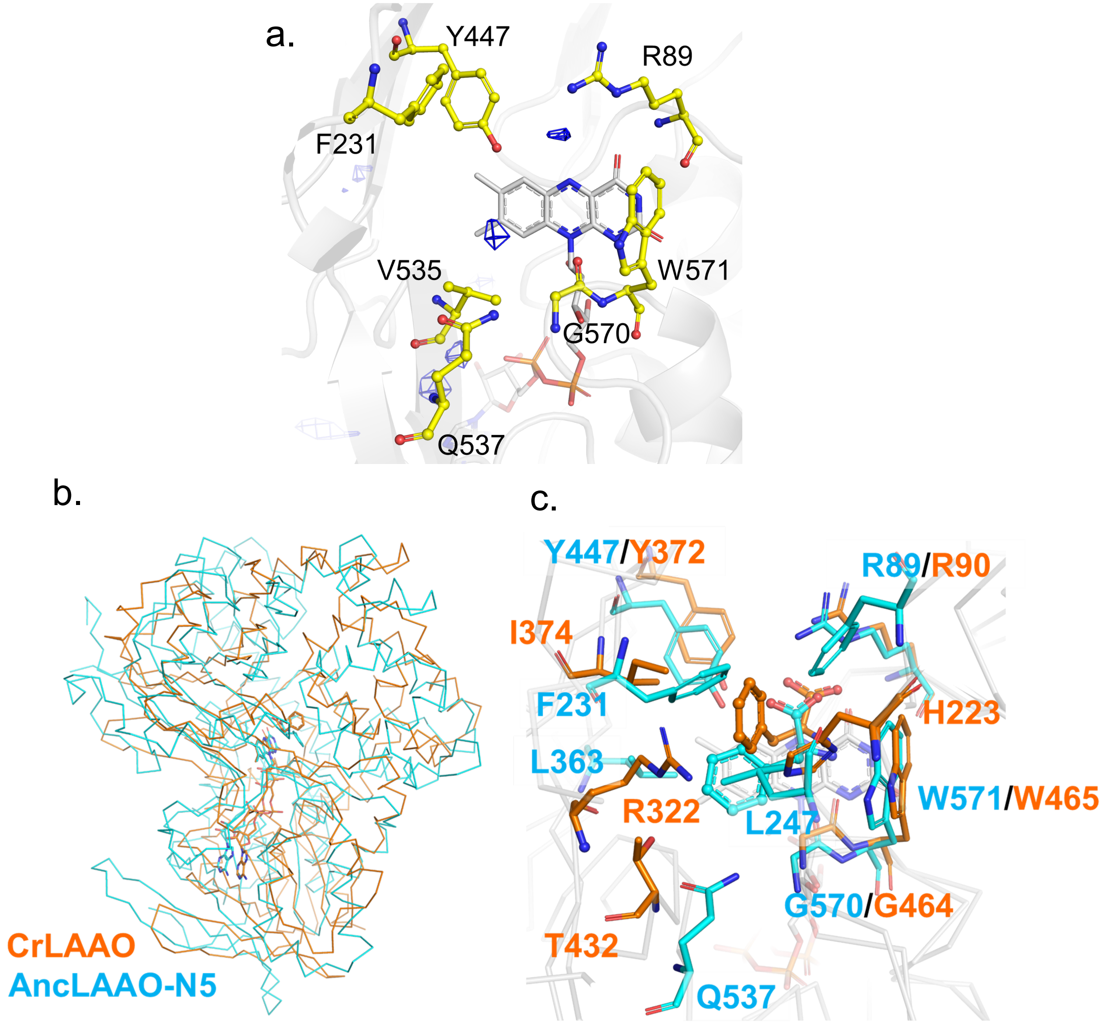

Supplementary Figure 8 a). Active site structure of ligand-free form of AncLAAO-N5. The polder F_o-F_c omit map was contoured at 4.0 σ, suggesting that no ligand is bound to the active site as expected. **b). Structural comparison between AncLAAO-N5 and LAAO from *Calloselasma rhodostoma* (CrLAAO, PDB ID: 2IID).** Structures for AncLAAO-N5 and CrLAAO were represented as orange and cyan, respectively. The root mean square deviation values for Cα atoms were 2.4 Å. **c). Structure comparison at active site of AncLAAO-N5 (cyan) and CrLAAO (orange).** Residues to recognize main chain of L-amino acids were highly conserved in the structures (R89, Y447, G570 and W571 in AncLAAO-N5). On the other hand, residues which would form interaction with side chain of L-amino acids were different in each other.

A.

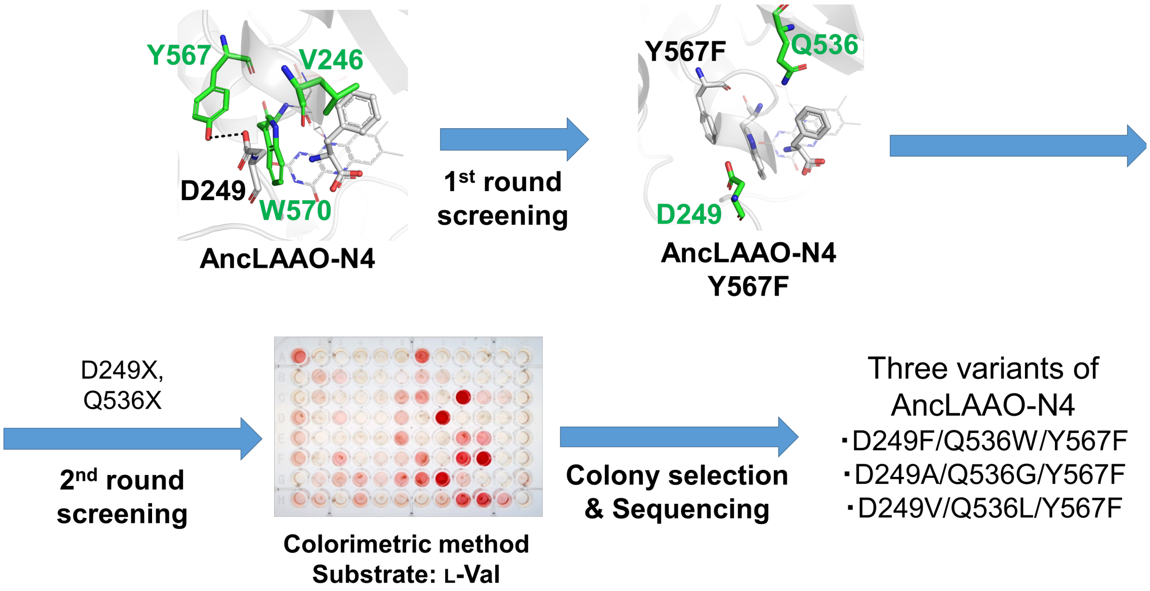

B.

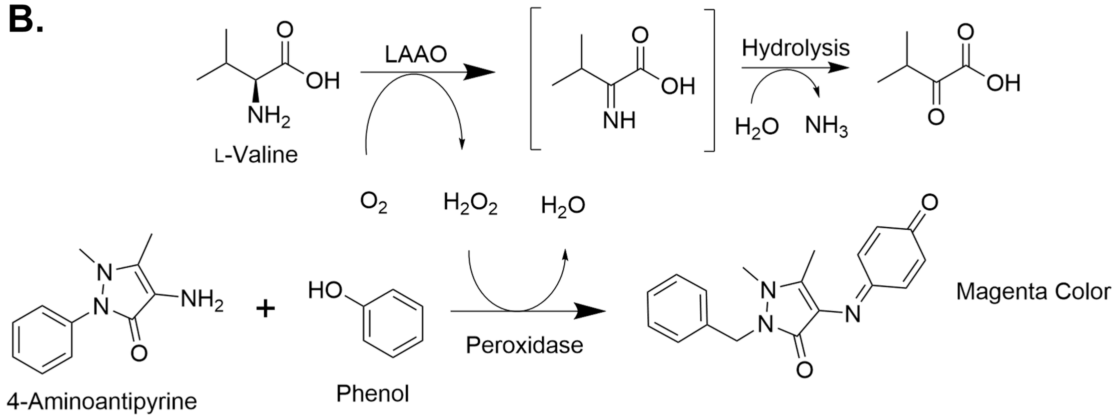

Supplementary Figure 9 A). Schematic view to screen AncLAAO-N4 variants which can oxidize L-Val more efficiently than wild type. For the 1st round screening, we can obtain AncLAAO-N4(Y567F) variant. Through secondary screening by colorimetric method, we succeeded in obtaining three AncLAAO-N4 variants: D249F/Q536W/Y567F, D249A/Q536G/Y567F, and D249V/Q536L/Y567F. **B). Reaction scheme of the colorimetric method to detect LAAO activity toward L-Val.** The scheme was proposed with referring to previous study (*2*).

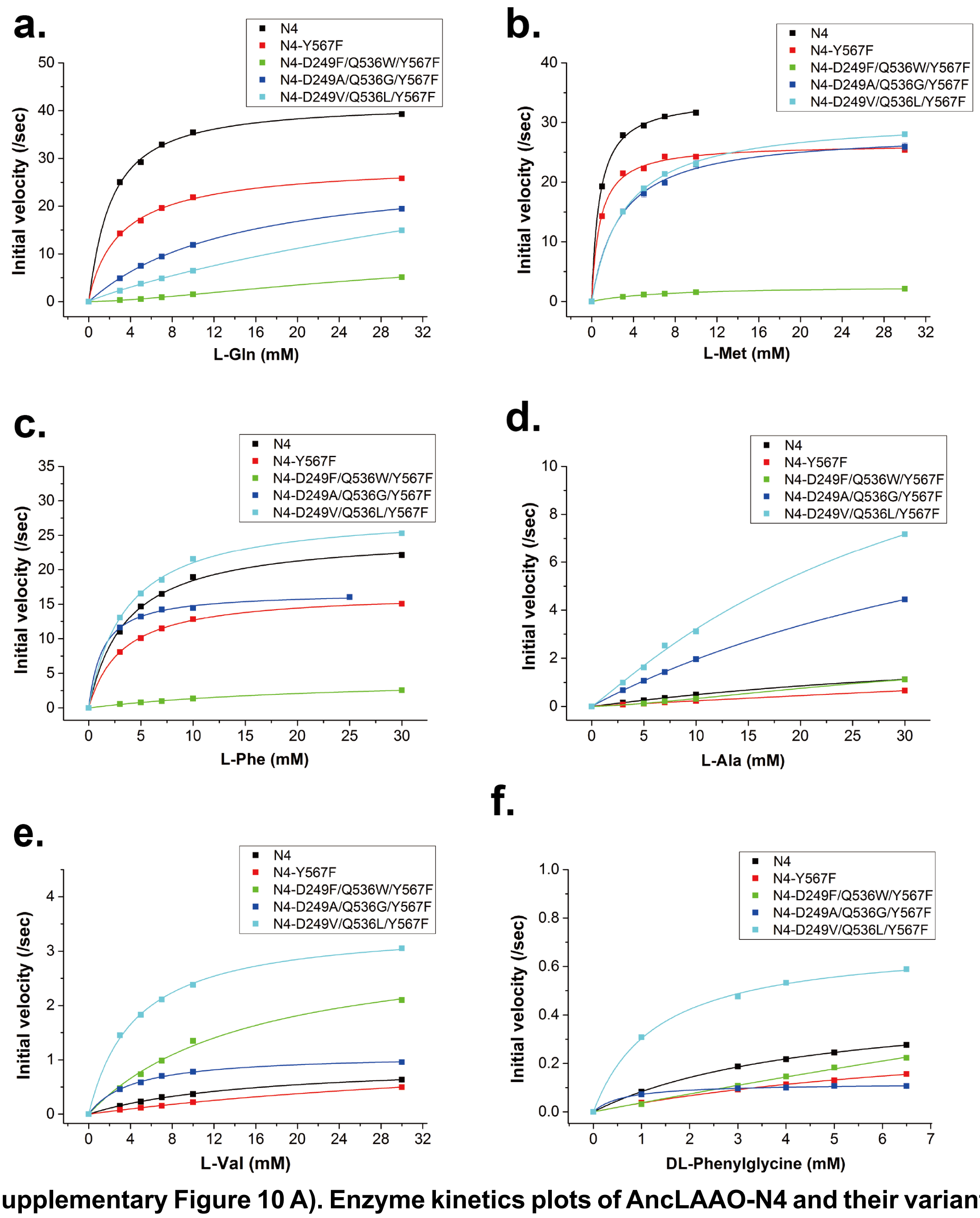

Supplementary Figure 10 A). Enzyme kinetics plots of AncLAAO-N4 and their variants toward L-Gln (a), L-Met (b), L-Phe (c), L-Ala (d), L-Val (e), and D,L-Phenylglycine (f), respectively. The initial velocities of AncLAAO-N4, Y567F, D249F/Q536W/Y567F, D249A/Q536G/Y567F, and D249V/Q536L/Y567F variants were represented as black, red, green, blue and cyan, respectively. The kinetics parameters were listed in Supplementary Table 7.

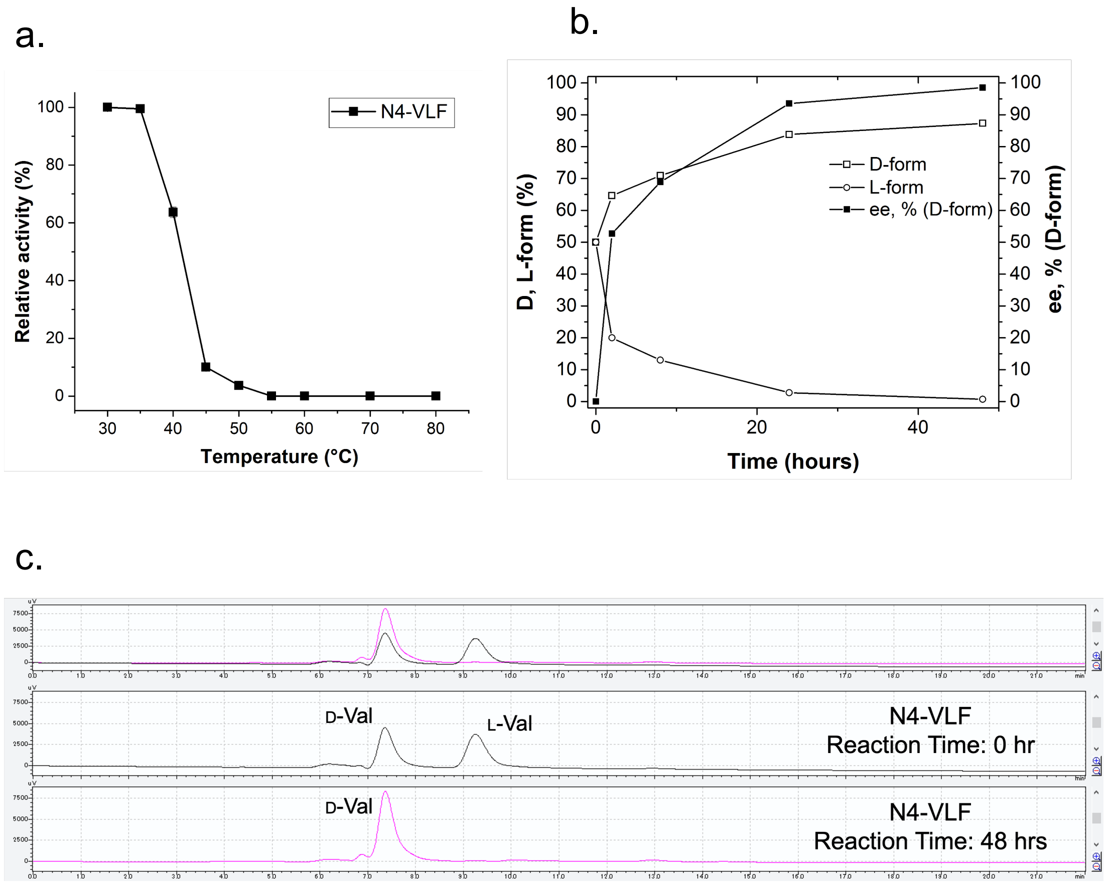

Supplementary Figure 11. Evaluation of short-time thermal stability of N4-VLF variant (a). Activity was measured after incubation of the samples for 10 min at different temperature. Relative activity was calculated by regarding the highest activity of each samples as 100%. **Time course of D-Val production from D,L-Val by deracemization reaction (b) and chiral HPLC chromatograms before (black line) and after deracemization (magenta) of racemic Valine by AncLAAO-N4(D249V/Q536L/Y568F) variant (c).** The retention time of D- and L-Val was 7.37 and 9.26 min, respectively. Running buffer condition was 0.92 % (w/v) $HClO_4$ and 20% acetonitrile, and flow rate and oven temperature were 0.15 mL/min and 20 ºC, respectively. The reactions were performed at preparative scale: Total 117 mg of D,L-Val was applied to the deracemization. Total 10 mg of the purified AncLAAOs variant was utilized in the reaction.

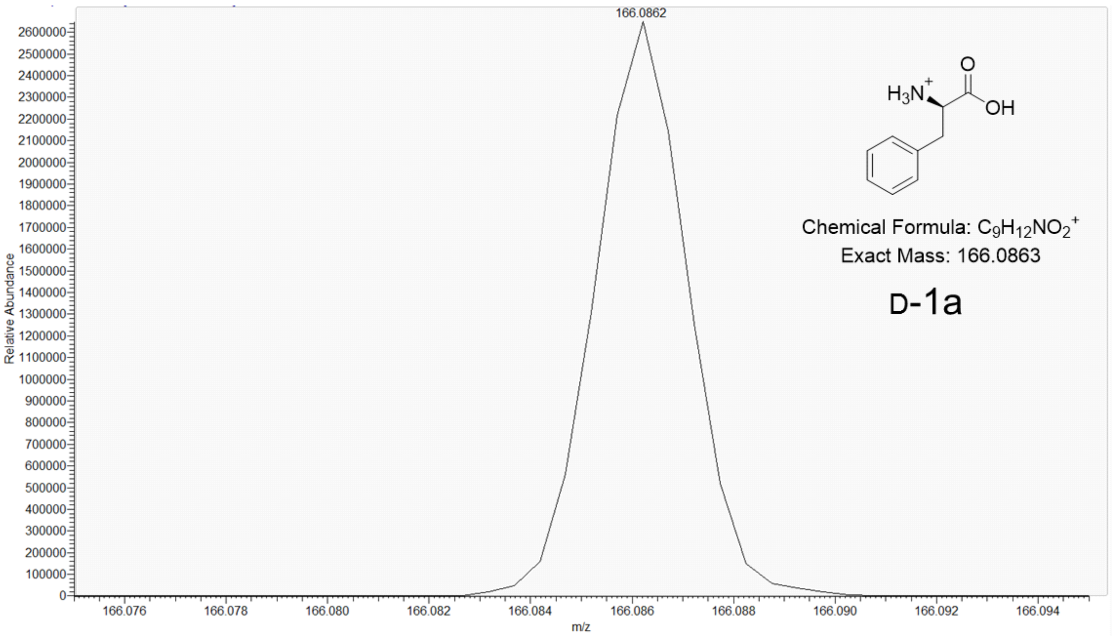

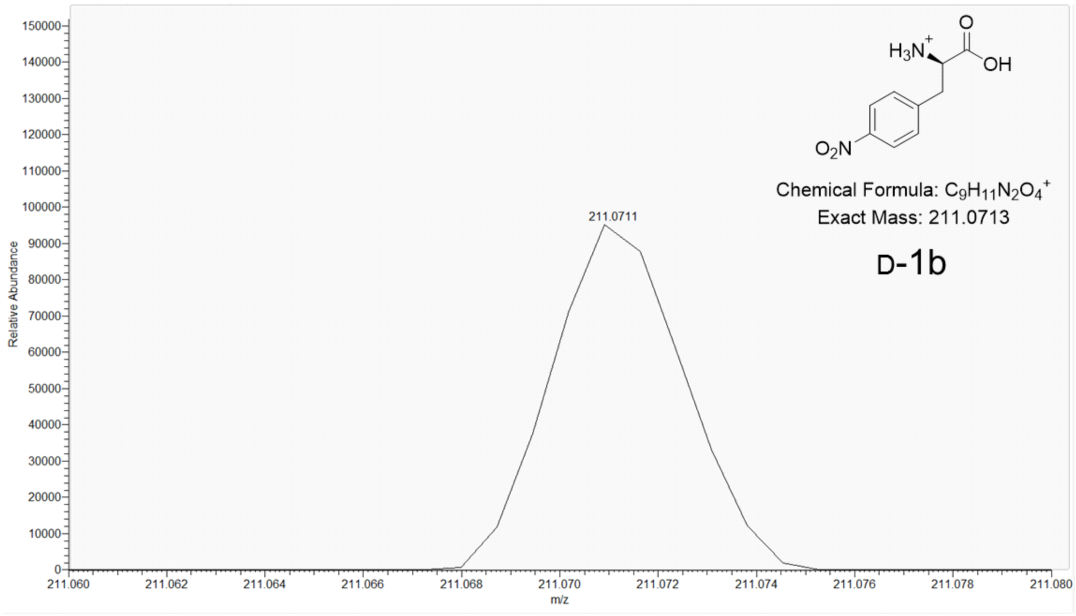

Supplementary Figure 12. HRMS analysis for products of the deracemization and stereoinversion reaction by AncLAAO-N4 (D-1a-1h). The observed positive ion spectrum for the products (Supplementary Figure 4-6, the peak area at D-**1a** to **1h**) were shown.

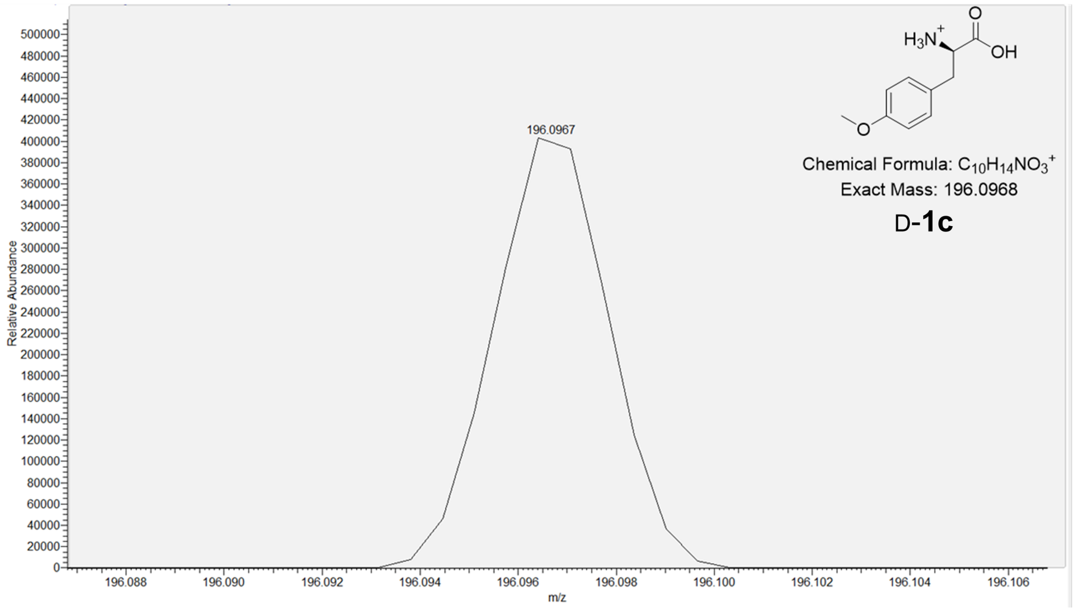

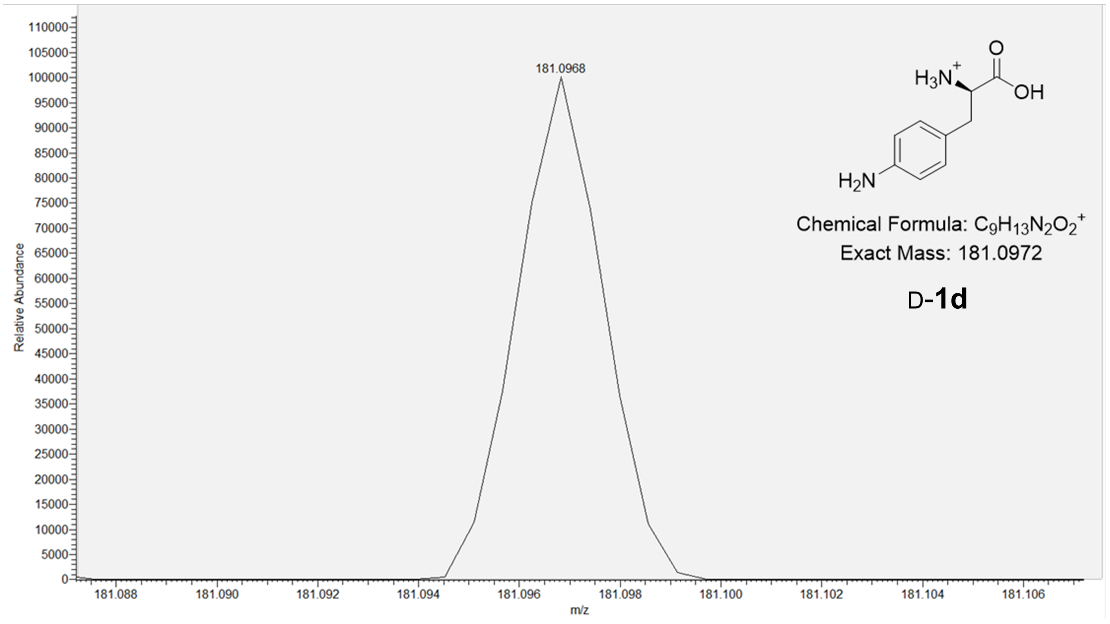

Supplementary Figure 12, Continue

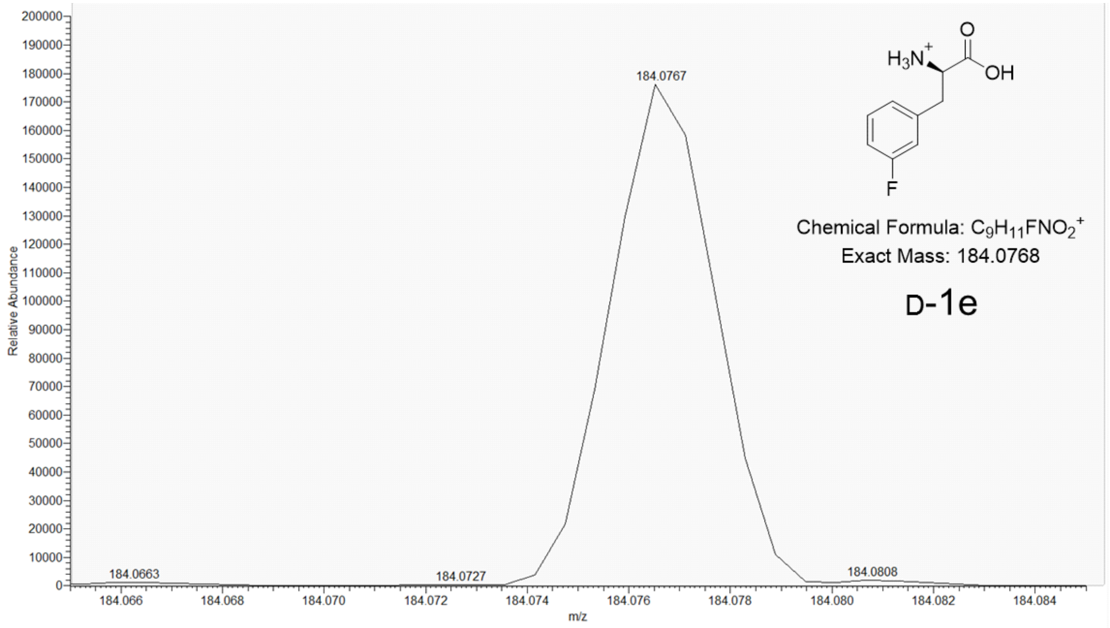

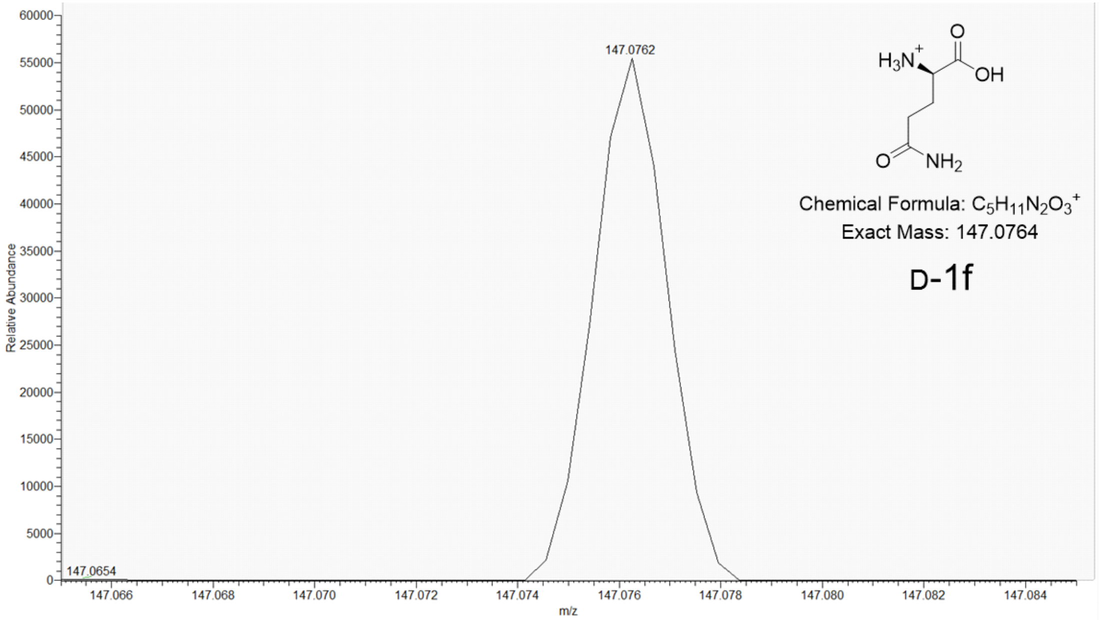

Supplementary Figure 12, Continue

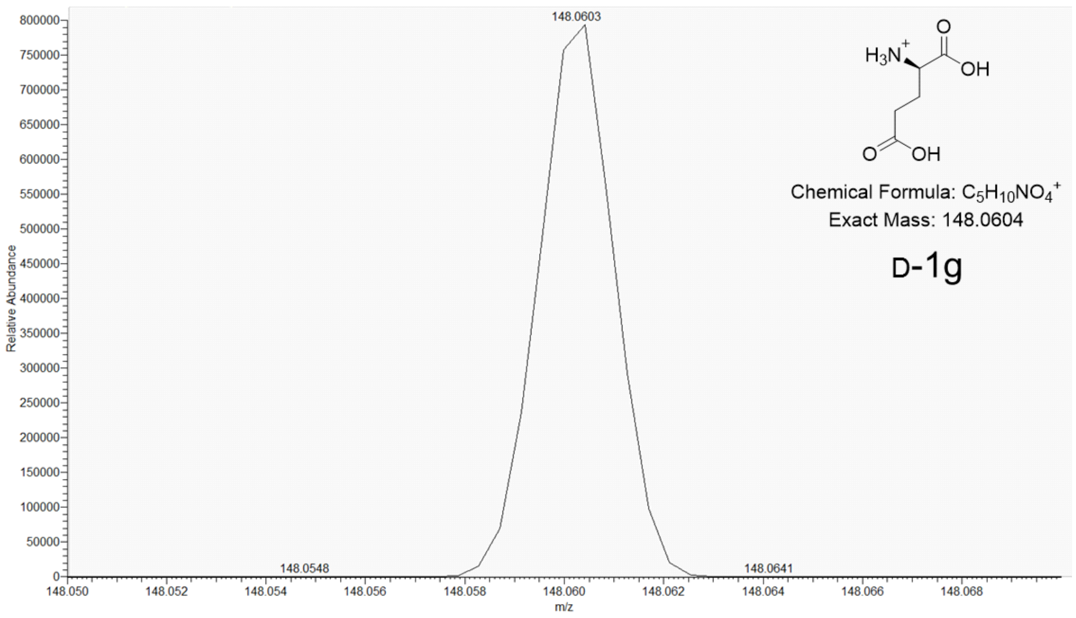

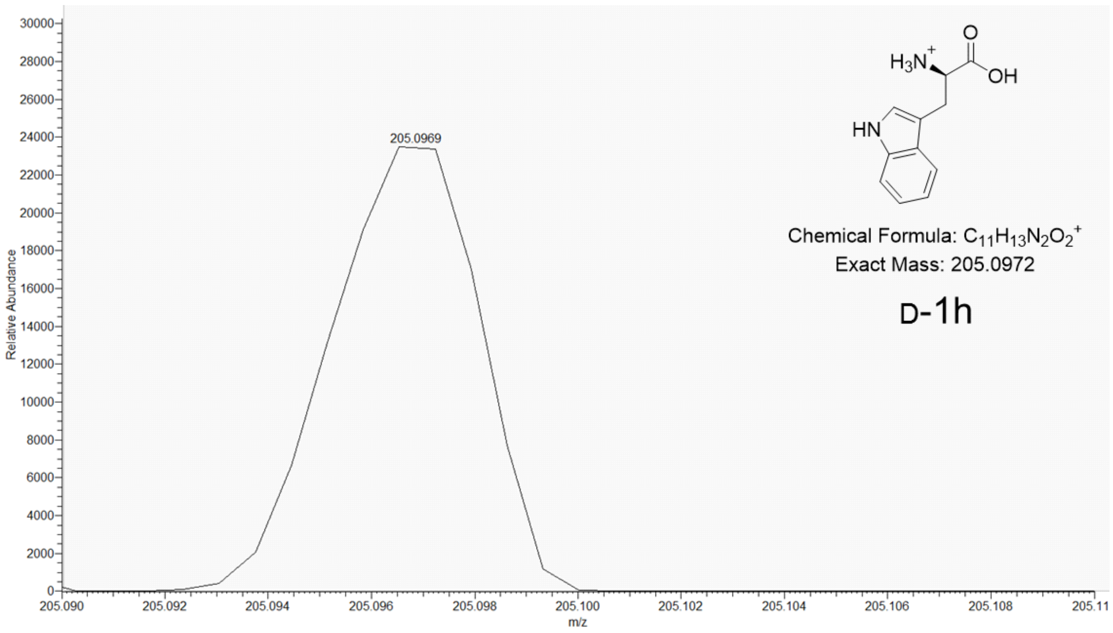

Supplementary Figure 12, Continue

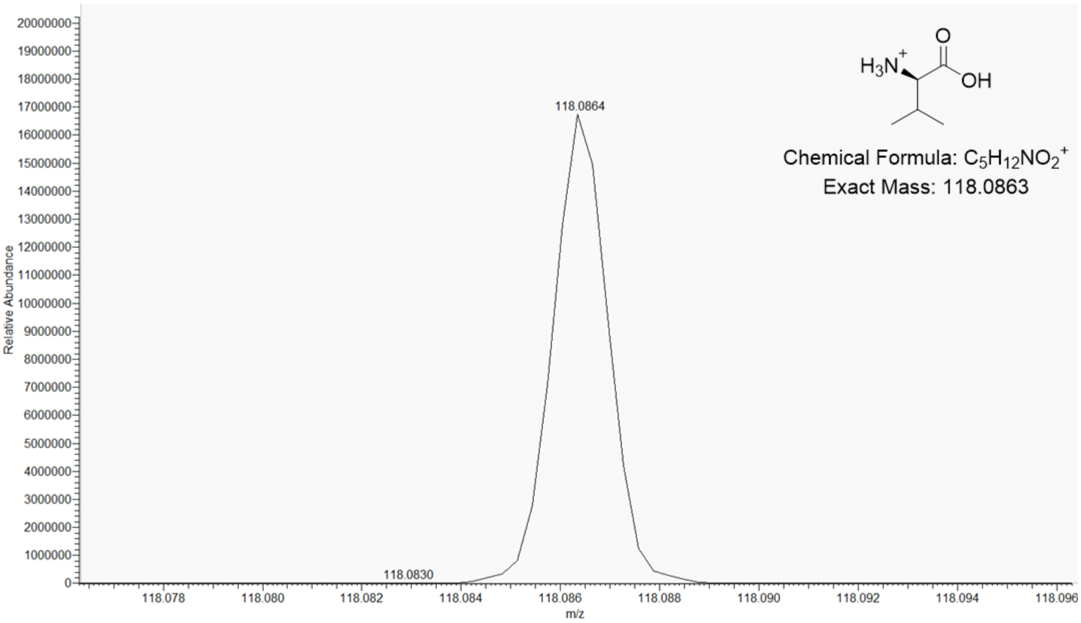

Supplementary Figure 12, Continue

Supplementary Table 1. Sequence identity between AncLAAOs and AROD

	AncLAAO-N1	AncLAAO-N2	AncLAAO-N3	AncLAAO-N4	AncLAAO-N5	AncAROD
AncLAAO-N1	100%	99.5%	92.3%	87.4%	82.7%	13.9%
AncLAAO-N2	99.5%	100%	92.5%	87.4%	82.3%	13.9%
AncLAAO-N3	92.3%	92.5%	100%	93.7%	76.4%	14.8%
AncLAAO-N4	87.4%	87.4%	93.7%	100%	73.3%	16.0%
AncLAAO-N5	82.7%	82.3%	76.4%	73.3%	100%	13.9%
AncAROD	13.9%	13.9%	14.8%	16.0%	13.9%	100%

Supplementary Table 2. Summary for purification of AncLAAOs from 1L cultivation after HisTrap-HP purification.

**Samples**	**Total protein (mg/L)**	**Total activity (U/L)**	**Specific activity (U/mg)**
AncLAAO-N1	50.7	651	12.8
AncLAAO-N4	55.1	247	4.5
AncLAAO-N4 (D249V/Q536L/Y568F)	40.1	252	5.3
AncLAAO-N5	78.4	166	2.1

Supplementary Table 3. Relative activity of AncLAAO-N1, AncLAAO-N4, and AncLAAO-N4 (D249V/Q536L/Y568F) toward 20 L-amino acids[a]

	AncLAAO-N1	AncLAAO-N4	AncLAAO-N4 (D249V/Q536L/Y568F)
Substrate	Relative activity (%)		
L-Glutamine	101.0 ± 1.8	100.0 ± 1.0	31.6 ± 0.1
L-Leucine	85.2 ± 1.7	92.8 ± 0.7	89.9 ± 0.7
L-Methionine	100 ± 2.1	92.7 ± 0.9	100 ± 0.9
L-Phenylalanine	52.9 ± 0.3	47.0 ± 0.7	97.1 ± 1.5
L-Glutamic acid	65.9 ± 1.4	41.7 ± 0.5	6.8 ± 1.1
L-Tryptophan	40.8 ± 0.5	38.2 ± 0.3	53.0 ± 1.4
L-Tyrosine	25.8 ± 1.1	11.2 ± 0.4	24.8 ± 0.4
L-Isoleucine	8.8 ± 0.2	4.1 ± 0.3	36.6 ± 0.6
L-Arginine	5.5 ± 0.2	2.8 ± 0.2	< 0.0
L-Histidine	< 0.0	1.1 ± 0.2	4.3 ± 0.8
L-Alanine	1.3 ± 0.4	0.98 ± 0.02	< 0.0
L-Valine	1.3 ± 0.4	0.73 ± 0.02	11.0 ± 0.1
L-Lysine	0.8 ± 0.6	0.24 ± 0.03	< 0.0
L-Proline	< 0.0	< 0.0	< 0.0
L-Cysteine	< 0.0	< 0.0	< 0.0
L-Aspartic Acid	< 0.0	< 0.0	< 0.0
Glycine	< 0.0	< 0.0	< 0.0
L-Asparagine	< 0.0	< 0.0	< 0.0
L-Serine	< 0.0	< 0.0	< 0.0
L-Threonine	< 0.0	< 0.0	< 0.0

[a] There is no activity toward 20 of D-amino acids. The relative activity toward L- and D-Tyr was measured utilizing substrate saturated solution.

Supplementary Table 4. Liquid chromatography (LC) condition and retention time for D,L-amino acid derivatives (D,L-1a-h)[a]

Compound	Flow rate (mL/min)	Oven temperature (ºC)	Retention time (min)	
			D-enantiomer	L-enantiomer
D,L-**1a**	0.5	30	5.00	8.00
D,L-**1b**	0.5	30	7.68	11.4
D,L-**1c**	0.5	30	7.22	11.4
D,L-**1d**	0.5	30	6.82	8.54
D,L-**1e**	0.5	30	5.70	9.01
D,L-**1f**	0.3	20	3.27	4.62
D,L-**1g**	0.3	20	3.72	7.98
D,L-**1h**	0.8	30	9.45	16.1

[a]The LC was performed by reverse phase HPLC on CROWNPAK-CR-I(+) column (150 mm × 3.0 mm × 5 µm, Daicel). Running buffer condition was 1.15% (w/v) $HClO_4$ for D,L-**1a** to **1e** and **1h**, and 0.98% (w/v) $HClO_4$ and 20% acetonitrile for D,L-**1f** and **1g**.

Supplementary Table 5. Top 10 of structures which have structural similarity to AncLAAO-N5 by Dali search.

Organism	Class	PDB code monomer	Z-score[a]	r.m.s.d.[b]	lali[c]	%id[d]
Calloselasma rhodostoma	L-Amino acid oxidase	2IID-B	30.6	2.4	440	19
Hypocrea rufa	L-Lysine oxidase	3X0V-A	28.1	2.6	448	18
Chromobacterium violaceum	L-Tryptophan oxidase	6ESE-A	27.4	2.5	390	20
Homo sapiens	Amine oxidase	2Z5X-A	25.5	3.1	433	15
Homo sapiens	Amine oxidase	6FVZ-A	24.8	3.3	435	14
Exiguobacterium sibiricum	Protoporphyrinogen Oxidase	3LOV-A	23.7	3.3	382	16
Pseudomonas savastanoi	Tryptophan 2-monooxigenase	4IV9-A	23.6	2.7	428	18
Pseudomonas sp. AIU 813	L-Amino acid oxidase/monooxigenase	5YB6-A	23.0	2.8	427	19
Homo sapiens	Lysine-specific histone demethylase 1	2X0L-A	22.7	2.9	395	18
Homo sapiens	Renalase	3QJ4-A	22.5	2.9	316	17

[a.] A measure of the statistical significance of the result relative to alignment of random structure.

[b.] Root mean square deviation for Cα atoms.

[c.] Number of aligned residues.

[d.] Sequence identity between AncLAAO-N5 and the targeted chain.

Supplementary Table 6. Primer list to prepare site-directed variants of AncLAAO-N4.

Primers	
R88A	5'- GGTGGCATG**GCT**TTCCTGTTTGACGGTATG-3'
Y446F	5'-CTGACCATC**TTC**TGCGATTATCTGAACATT-3'
Y567F	5'-TTTAGCGAC**TTC**CAAGGTTGGGTTGAAGGT-3'
L246X/D249X	5'-GCGTATCAG**NNS**CTGGAG**NNS**TTCCCGGCGGGTGTT-3'
Q536X	5'-GGCGTGCAC**NNS**TGGGCGGTTGGTGCGAAC-3'

Supplementary Table 7. Enzyme kinetic parameters of AncLAAO-N4 and their variants toward L-Met, L-Gln, L-Phe, L-Val, L-Ala and D,L-Phenylglycine[a].

	AncLAAO-N4			AncLAAO-N4 (Y567F)			AncLAAO-N4 (D249F/Q536W/Y567F)		
Compounds	k_{cat}	K_m	k_{cat}/K_m	k_{cat}	K_m	k_{cat}/K_m	k_{cat}	K_m	k_{cat}/K_m
	/sec	mM	/(sec·mM)	/sec	mM	/(sec·mM)	/sec	mM	/(sec·mM)
L-Met	34.2 ± 0.3	0.76 ± 0.04	45.0	26.4± 0.4	0.80 ± 0.07	33.0	2.6 ± 0.1	6.9 ± 0.3	0.38
L-Gln	42.3 ± 0.5	2.1 ± 0.1	20.1	28.5± 0.5	3.2 ± 0.2	8.9	n.d.	n.d.	0.33
L-Phe	25.1 ± 0.5	3.6 ± 0.2	7.0	16.7 ± 0.1	3.2 ± 0.1	5.2	4.6 ± 0.2	23.9 ± 2.0	0.19
L-Val	1.0 ± 0.0	15.6 ± 0.9	0.06	1.4 ± 0.1	52.9 ± 5.0	0.03	3.2 ± 0.2	15.7 ± 1.8	0.20
L-Ala	3.3 ± 0.1	58.9 ± 2.8	0.06	n.d.	n.d.	0.02	n.d.	n.d.	0.07
D,L-Phenylglycine	0.47 ± 0.01	4.7 ± 0.2	0.10	0.38 ± 0.02	9.3 ± 0.6	0.04	n.d.	n.d.	0.04

	AncLAAO-N4 (D249A/Q536G/Y567F)			AncLAAO-N4 (D249V/Q536L/Y567F)		
Compounds	k_{cat}	K_m	k_{cat}/K_m	k_{cat}	K_m	k_{cat}/K_m
	/sec	mM	/(sec·mM)	/sec	mM	/(sec·mM)
L-Met	28.5 ± 0.6	2.8 ± 0.2	10.2	30.9 ± 0.3	3.2 ± 0.1	9.7
L-Gln	28.8 ± 0.2	14.3 ± 0.2	2.0	39.9 ± 1.6	50.4 ± 2.9	0.79
L-Phe	16.7 ± 0.2	1.3 ± 0.1	12.8	28.5 ± 0.5	3.6 ± 0.2	7.9
L-Val	1.1 ± 0.0	4.2 ± 0.2	0.26	3.5 ± 0.1	4.5 ± 0.2	0.78
L-Ala	12.2 ± 0.2	52.4 ± 1.0	0.23	19.6 ± 1.9	51.9 ± 7.0	0.38
D,L-Phenylglycine	0.12 ± 0.0	0.63 ± 0.05	0.19	0.70 ± 0.0	1.3 ± 0.1	0.54

[a] The measurement of enzyme kinetic parameters was performed independently six times (N = 6).

Supplementary References

1. Katoh, K., and Toh, H. (2008) Recent developments in the MAFFT multiple sequence alignment program, *Brief Bioinform 9*, 286-298.
2. Parmeggiani, F., Lovelock, S. L., Weise, N. J., Ahmed, S. T., and Turner, N. J. (2015) Synthesis of D- and L-phenylalanine derivatives by phenylalanine ammonia lyases: a multienzymatic cascade process, *Angew Chem Int Ed Engl 54*, 4608-4611.
3. Robert, X., and Gouet, P. (2014) Deciphering key features in protein structures with the new ENDscript server, *Nucleic Acids Res 42*, W320-324.
