# Supplementary material for: Ancestral L-amino acid oxidases for deracemization and stereoinversion of amino acids
Source: Commun Chem. 2020 Dec 4;3:181. doi: 10.1038/s42004-020-00432-8 (PMC9814856; doi:10.1038/s42004-020-00432-8)
Supplement: Supplementary file 3 — Description of Additional Supplementary Files [file 42004_2020_432_MOESM3_ESM.pdf]

Description of Additional Supplementary Files

File Name: Supplementary Data 1
Description: Protein sequences of five ancestral L-amino acid oxidases:
AncLAAO-N1, AncLAAO-N2, AncLAAO-N3, AncLAAO-N4 and AncLAAO-N5

File Name: Supplementary Data 2
Description: Nucleotide sequences of three ancestral L-amino acid oxidases:
AncLAAO-N1, AncLAAO-N4 and AncLAAO-N5

File Name: Supplementary Data 3
Description: Validation report of 7C4K

File Name: Supplementary Data 4
Description: Validation report of 7C4L

File Name: Supplementary Data 5
Description: Validation report of 7C4M

File Name: Supplementary Data 6
Description: Validation report of 7C4N
