# Supplementary material for: Ancestral L-amino acid oxidases for deracemization and stereoinversion of amino acids
Source: Commun Chem. 2020 Dec 4;3:181. doi: 10.1038/s42004-020-00432-8 (PMC9814856; doi:10.1038/s42004-020-00432-8)
Supplement: Supplementary file 4 — Supplementary Data 1 [file 42004_2020_432_MOESM4_ESM.docx]

| >AncLAAO-N1 |
| --- |
| MTHYKFGNEISDKSIPKQVKVAIVGAGMSGLYSAWRLQNEANTQDLAIFERSDRTGGRLDSDLIEFKNQRSGPETPSTITVKEEQGGMRFLFEGMDDLMALFLKLDLQDQIVPFPMNSGGNNRLYFRGESFSVNDAQQDDYAIWSHLYNLDPSEQGVNPKDIINVVFNRILQANPQFDARPEVRGPEFWQSFRLECQWQGKTLNEWTLWDLFTDMGYSQECITMLYRVLGFNGTFLSKMNAGVAYQLLEDFPADVQFKTFKDGFSTLPNALVDKIGTDKIHLQTSIEEIDFDEASGKYVLHYTHTDEHGQVHKGQVKAEKVILGLPRLALEKLFVRSNAFNRLEKKRSEQLWNTLQSASNQPLLKINLYYDTAWWGRGITGRPAVEFGPNFADLPTGSVYPFYAVNDELAAALMYEERHTNPSQDTQHKLDGINSEKYERPAALTIYCDYLNINFWSALQNKGELYHHPHQDEYVESVPSDIYPASTAVVQQATKFFKDIFNTHYVPEPILTSARIWEGSVNFDVPASQQFGFGVHQWAVGANDKQVMEDLVEPLPNLFTCGEAFSDYQGWVEGALRSTDLVLEKGFGLAPLSEVYEQNTHISSSEAIKAVYEENSSKLINQYIDPNFSANTAPIEKLADVNSVIGVNLSYFDKP |
| >AncLAAO-N2  MTHYKFGNEISDKSIPKQVKVAIVGAGMSGLYSAWRLQNEANTQDLAIFERSDRTGGRLDSDLIEFKNQRSGPDTPKTITVKEEQGGMRFLFEGMDDLMALFLKLDLQDQIVPFPMNSGGNNRLYFRGESFSVNDAQQDDYAIWSHLYNLDPSEQGVNPKDIINVVFNRILQANPQFDARPEVRGPEFWQSFRLECQWQGKTLNEWTLWDLFTDMGYSQECITMLYRVLGFNGTFLSKMNAGVAYQLLEDFPADVQFKTFKDGFSTLPNALVDKIGTDKIHLQTSIEEIDFDEASGKYVLHYTHTDEHGQVHKGQVKAEKVILGLPRLALEKLFVRSNAFNRLEKKRSEQLWNTLQSASNQPLLKINLYYDTAWWGRGITGRPAVEFGPNFADLPTGSVYPFYAVNDELAAALMYEERDTNPSQDTQHKLDGINSEKYERPAALTIYCDYLNINFWSALQNKGELYHHPHQDEYVESVPSDIYPASTAVVQQATKFFKDIFNTHYVPEPILTSARIWEGSVNFDVPASQQFGFGVHQWAVGANDKQVMEDLVEPLPNLFTCGEAFSDYQGWVEGALRSTDLVLEKGFGLAPLSEVYEQNTHISSSEAIKAVYEENSSKLINQYIDPNFSANTAPIEKLADVNSVIGVNLSYFDKP |
| >AncLAAO-N3  MTHYTFGNEISDKSLPKQVKVAIVGAGMSGLYSAWRLQNEANCQDLAIFERSNRTGGRLDSDLIEFKNQRSDTPKTITVKEEQGGMRFLFDGMDDLMALFLKLDLQDQIVPFPMNSGGNNRLFFRGESFSVNDAQQDDYAIWSHLYNLDPSEQGVNPKDIVNVVFNRILQANPQFQARPEVRGPEFWQAFRLECQWQGKTLNEWTLWDLYTDMGYSQECINMLYRVLGFNGTFLSQMNAGVAYQLLEDFPAGVQFKTFKDGFSTLPNALVDKVGTDNIHLQTSIEEIDFAEESGHYSLHYSHTDEHGRVHKGQVKAEKVILGLPRLALEKLFVRSNAFNRLDKERSEQLWNTLQSASNQPLLKINLYYDTAWWGRGTTGRPAVEFGPNFADLPTGSVYPFYAVNDELAAALMYQERKTNPSQDTQAKLDRISSEKYERPAALTIYCDYLNINFWSALQNIGEIYHHPHQDDYVEDVPSDIYPASTAVVEQATRFFKDIFNTHYVPEPILTSARIWEGSVRFDVPASQQFGFGVHQWAVGANDKQVMADLTEPLPNLFTCGEAFSDYQGWVEGALRSTDLALEKGFGLKPLSEVYEQNTHISSSEAIKAVYEENSSKLINQYIEPNFSANTAPIEKPADVDSVIGVNLSYFDTK  >AncLAAO-N4  MGTHYTFGKEITDKPLPTQVKVAIVGAGMSGLYSAWRLQQEANCQDLAIFERSNRTGGRLDSDLIEFKNLRSETPKTITVKEEQGGMRFLFDGMDDLMALFLKLNLQDDIVPFPMNSGGNNRLFFRGESFSVEDAQQDDYAIWSHLYNLDQSEQGVNPKDIVNVVFNRILEANPQFQQRPEVRGPEFWQAFRLECQWQGQTLNEWTLWDLYTDMGYSQECINMLYRVLGFNGTFLSQMNAGVAYQLLEDFPAGVQFKTFKDGFSTLPNKLVEEVGTDNIHLQTSIEEIDFAEESGLYSLHYSHTDEHGRVHKGQVKAEKVILGLPRLALEKLFVRSNAFNRLDKDRSEQLWNTLQSASNQPLLKINLYYDSAWWGRGTTGRPAVEFGPNFADLPTGSVYPFYAVNDELAAALMYQERSTNPSKAVQAKLDRIGNEKYERPAALTIYCDYLNINFWSNLQNIGETYHHPHQDDYVEDVPADIYPASTAVVEQATRFFKDIFNTHYVPEPILTSARIWEGSVRFDIPASRQFGFGVHQWAVGANDKEVMATLAEPLPNLFTCGEAFSDYQGWVEGALRSTDLALEKGFGLKPLSQVYFENTNISSSDAIKAVYEENSSKLINQYIETNFSANTAPIEKTADVDSVIGVNLSYFDTK  >AncLAAO-N5  MTHYKLGSDISQKSIPKEVKVAIVGAGMSGLYSAWRLQSEANVGDLAIFERSDRTGGRLDSDLIEFKDNRAGAEPGSTITVKEEQGGMRFLFEGMDDLMALFLKLGLEDQIVPFPMNSGGNNRLYFRGTSFSVNDAEQDDYHIWSALYNLDPSEQGVNPKDIINVVFNRILQVNPQFDSRPEVRGPEFWQNFRLQCQWQGEPLYNWSLWDLLTDMGYSQECITMLYRVLGFNGTFLSKMNAGVAYQLLEDFPADVEFRTFKDGFSTLPNALVDKIGKDKIHLQTSIDSIAFDKADSKYVLKYTKIDQSGQVSEGKFKAEKVILGLPRLALEKLFIASDAFKQLPKKRRDELWDTLQSTSNQPLLKINLYYDTAWWGTGMTGRPAVSFGPNFADLPTGSVYPFYALNDELAAALMYDERHATPNPDTQHKLDGIDAAKYARPAALTIYCDYLNINFWSALQNKGELYHHPHESELVESIPSDIFPASEAVVQQATQFFKDIFNTHYVPQPTLTSARIWEGNVNFNVPENLQFGFGVHQWAIGANDKEVIEDLVEPLPNLFTCGEAYSDYQGWVEGALRSTDLVLQKGFGLAPLSEVYEQDQGRSSSEAIQIAYRKISNKMIMEYIDPNFSPNTKHKVTLAEVNSVLGVNLSYFDKP |
