# Supplementary material for: Ancestral L-amino acid oxidases for deracemization and stereoinversion of amino acids
Source: Commun Chem. 2020 Dec 4;3:181. doi: 10.1038/s42004-020-00432-8 (PMC9814856; doi:10.1038/s42004-020-00432-8)
Supplement: Supplementary file 5 — Supplementary Data 2 [file 42004_2020_432_MOESM5_ESM.docx]

>AncLAAO-N1_dna

ATGACACATTACAAATTTGGCAACGAAATCAGCGACAAGAGCATCCCGAAGCAAGTTAAAGTGGCAATTGTTGGCGCCGGCATGAGCGGTCTGTATAGCGCATGGCGCTTACAGAATGAGGCCAATACCCAAGATCTGGCAATCTTTGAGCGCAGCGATCGTACCGGCGGCCGCTTAGACAGTGATCTGATTGAGTTTAAAAATCAGCGCAGCGGCCCCGAAACCCCGAGCACCATCACCGTGAAGGAAGAGCAAGGTGGCATGCGTTTTCTGTTCGAGGGTATGGACGATTTAATGGCTTTATTTCTGAAATTAGATTTACAAGATCAGATCGTGCCGTTTCCGATGAATAGCGGCGGCAACAATCGTTTATATTTCCGCGGCGAGAGTTTCAGTGTGAACGATGCCCAGCAAGATGACTACGCAATTTGGAGCCATTTATACAATCTGGACCCGAGCGAGCAAGGTGTGAATCCGAAAGATATCATCAATGTGGTGTTTAACCGTATTTTACAAGCTAACCCGCAGTTTGATGCACGTCCGGAAGTTCGTGGTCCGGAGTTTTGGCAGAGCTTTCGTTTAGAGTGCCAATGGCAAGGTAAGACTTTAAACGAATGGACTTTATGGGATTTATTCACCGACATGGGCTATAGCCAAGAATGCATCACCATGCTGTATCGCGTGCTGGGTTTTAATGGCACCTTTTTAAGCAAAATGAACGCCGGTGTGGCCTATCAACTGCTGGAAGACTTCCCCGCTGATGTGCAGTTCAAGACCTTCAAGGACGGTTTTAGTACTTTACCGAATGCCTTAGTGGACAAGATCGGCACCGATAAGATTCATCTGCAAACCAGTATTGAAGAGATCGATTTCGATGAGGCCAGCGGCAAATACGTGTTACACTATACCCATACCGACGAGCATGGCCAAGTTCATAAGGGCCAAGTTAAAGCCGAGAAGGTGATTTTAGGTCTGCCTCGTCTGGCTTTAGAAAAACTGTTCGTGCGCAGCAACGCCTTCAATCGTCTGGAAAAGAAGCGTAGCGAACAGCTGTGGAACACTTTACAGAGCGCCAGCAATCAGCCGCTGCTGAAAATCAATTTATACTACGACACCGCATGGTGGGGTCGTGGCATTACCGGCCGTCCGGCAGTGGAATTCGGTCCGAACTTCGCAGATTTACCGACTGGTAGCGTGTACCCGTTTTACGCCGTGAATGACGAACTGGCAGCCGCCTTAATGTACGAGGAGCGTCATACCAATCCGAGCCAAGATACCCAGCACAAGTTAGATGGCATCAATAGTGAAAAGTACGAGCGTCCGGCCGCTTTAACCATTTATTGTGATTACTTAAATATTAACTTCTGGAGTGCTTTACAGAACAAAGGCGAGCTGTACCATCATCCGCACCAAGATGAATACGTGGAGAGCGTGCCGAGCGATATCTATCCGGCCAGCACCGCAGTGGTGCAGCAAGCTACCAAATTCTTTAAAGACATCTTTAATACCCACTATGTGCCGGAGCCTATTCTGACCAGCGCACGTATTTGGGAGGGCAGCGTGAATTTCGATGTGCCCGCTAGCCAGCAGTTTGGCTTTGGTGTTCACCAGTGGGCAGTGGGTGCCAATGACAAACAAGTTATGGAGGATTTAGTTGAACCGCTGCCGAATCTGTTTACTTGTGGCGAAGCCTTCAGCGATTACCAAGGTTGGGTGGAGGGTGCTTTACGCAGCACAGATTTAGTTCTGGAGAAAGGTTTTGGTCTGGCACCGCTGAGTGAAGTGTATGAACAGAACACCCATATTAGCAGCAGCGAAGCCATTAAAGCCGTGTACGAGGAGAATAGTAGCAAACTGATCAATCAGTATATTGACCCGAACTTTAGCGCCAATACCGCCCCGATTGAGAAATTAGCCGATGTGAACAGCGTTATCGGTGTTAATTTAAGCTATTTCGATAAACCT

>AncLAAO-N4_dna

ATGGGCACCCACTACACCTTCGGCAAGGAAATCACCGACAAACCGCTGCCGACCCAAGTGAAGGTTGCGATTGTGGGTGCGGGCATGAGCGGCCTGTATAGCGCGTGGCGTCTGCAGCAAGAGGCGAACTGCCAGGATCTGGCGATCTTCGAACGTAGCAACCGTACCGGTGGCCGTCTGGACAGCGATCTGATCGAGTTTAAGAACCTGCGTAGCGAAACCCCGAAGACCATTACCGTTAAAGAGGAACAAGGTGGCATGCGTTTCCTGTTTGACGGTATGGACGATCTGATGGCGCTGTTCCTGAAACTGAACCTGCAGGACGATATCGTGCCGTTTCCGATGAACAGCGGTGGCAACAACCGTCTGTTCTTTCGTGGTGAGAGCTTCAGCGTTGAAGATGCGCAGCAAGACGATTACGCGATTTGGAGCCACCTGTATAACCTGGACCAAAGCGAACAGGGCGTGAACCCGAAAGATATCGTTAACGTGGTTTTCAACCGTATTCTGGAGGCGAACCCGCAATTTCAGCAACGTCCGGAAGTGCGTGGTCCGGAATTCTGGCAGGCGTTTCGTCTGGAGTGCCAGTGGCAAGGCCAGACCCTGAACGAATGGACCCTGTGGGACCTGTACACCGATATGGGTTATAGCCAGGAATGCATCAACATGCTGTACCGTGTTCTGGGTTTCAACGGCACCTTTCTGAGCCAAATGAACGCGGGCGTGGCGTATCAGCTGCTGGAGGACTTCCCGGCGGGTGTTCAATTCAAGACCTTTAAAGATGGCTTTAGCACCCTGCCGAACAAACTGGTGGAGGAAGTGGGTACCGACAACATCCACCTGCAGACCAGCATCGAGGAAATTGATTTCGCGGAGGAAAGCGGCCTGTACAGCCTGCACTATAGCCACACCGACGAGCACGGTCGTGTTCACAAAGGCCAAGTGAAGGCGGAAAAAGTTATTCTGGGTCTGCCGCGTCTGGCGCTGGAGAAGCTGTTCGTGCGTAGCAACGCGTTTAACCGTCTGGACAAAGATCGTAGCGAACAGCTGTGGAACACCCTGCAAAGCGCGAGCAACCAGCCGCTGCTGAAGATTAACCTGTACTATGACAGCGCGTGGTGGGGTCGTGGTACCACCGGTCGTCCGGCGGTGGAGTTTGGTCCGAACTTTGCGGACCTGCCGACCGGCAGCGTGTACCCGTTCTATGCGGTTAACGATGAGCTGGCGGCGGCGCTGATGTACCAAGAACGTAGCACCAACCCGAGCAAGGCGGTTCAGGCGAAACTGGACCGTATCGGTAACGAGAAATATGAACGTCCGGCGGCGCTGACCATCTACTGCGATTATCTGAACATTAACTTTTGGAGCAACCTGCAAAACATTGGCGAAACCTACCACCACCCGCACCAGGACGATTATGTGGAGGACGTTCCGGCGGATATCTATCCGGCGAGCACCGCGGTGGTTGAGCAGGCGACCCGTTTCTTTAAGGACATTTTCAACACCCACTATGTGCCGGAGCCGATCCTGACCAGCGCGCGTATTTGGGAAGGTAGCGTTCGTTTTGACATCCCGGCGAGCCGTCAATTCGGTTTTGGCGTGCACCAGTGGGCGGTTGGTGCGAACGATAAAGAGGTGATGGCGACCCTGGCGGAACCGCTGCCGAACCTGTTCACCTGCGGCGAGGCGTTTAGCGACTACCAAGGTTGGGTTGAAGGTGCGCTGCGTAGCACCGATCTGGCGCTGGAAAAGGGTTTCGGCCTGAAACCGCTGAGCCAGGTGTACTTTGAGAACACCAACATCAGCAGCAGCGACGCGATTAAGGCGGTTTACGAGGAAAACAGCAGCAAACTGATCAACCAGTATATTGAGACCAACTTCAGCGCGAACACCGCGCCGATCGAAAAGACCGCGGACGTGGATAGCGTTATTGGTGTGAACCTGAGCTACTTTGATACCAAA

>AncLAAO-N5_dna

ATGACCCATTACAAGCTGGGCAGCGATATCAGCCAAAAGAGCATTCCGAAAGAAGTGAAAGTTGCCATCGTGGGCGCCGGCATGAGCGGTCTGTACAGCGCTTGGCGTTTACAGAGCGAAGCCAATGTGGGCGATCTGGCCATCTTTGAGCGTAGCGATCGCACGGGCGGTCGTCTGGACAGCGATCTGATCGAATTCAAAGATAATCGCGCCGGCGCCGAGCCCGGTAGCACGATTACCGTGAAGGAAGAACAAGGTGGTATGCGCTTTTTATTTGAGGGTATGGATGATCTGATGGCTTTATTTTTAAAGCTGGGTTTAGAGGACCAAATCGTGCCATTTCCGATGAACAGCGGTGGTAACAACCGTTTATACTTTCGCGGCACCAGCTTTAGCGTGAATGATGCCGAGCAAGATGACTATCACATCTGGAGCGCGCTGTACAACCTCGATCCAAGCGAACAAGGTGTGAATCCAAAAGACATCATCAACGTGGTGTTTAATCGCATTTTACAAGTTAACCCACAGTTTGATAGCCGCCCGGAAGTTCGTGGCCCGGAATTCTGGCAGAACTTTCGCCTCCAGTGCCAGTGGCAAGGTGAACCGCTGTACAACTGGAGTCTGTGGGATCTGCTGACCGATATGGGCTACAGCCAAGAATGTATCACCATGCTCTACCGCGTTCTCGGCTTCAATGGCACCTTTCTGAGCAAGATGAACGCCGGTGTTGCCTACCAACTGCTGGAGGACTTTCCGGCCGACGTGGAGTTTCGCACCTTCAAGGACGGCTTTAGCACTTTACCGAATGCTTTAGTGGATAAAATTGGCAAAGATAAAATCCATTTACAGACGAGCATCGACAGCATCGCGTTCGACAAGGCCGACAGCAAATATGTGCTGAAATATACCAAAATCGATCAGAGTGGCCAAGTTAGCGAGGGCAAATTTAAAGCCGAAAAGGTGATTCTGGGTCTGCCACGCCTCGCTTTAGAGAAGCTGTTTATCGCGAGCGACGCCTTTAAGCAACTCCCGAAAAAACGTCGCGACGAACTCTGGGATACTTTACAGAGTACGAGTAACCAGCCGCTGCTCAAAATCAACCTCTACTACGATACGGCGTGGTGGGGTACCGGTATGACCGGTCGCCCGGCCGTTAGCTTTGGCCCGAATTTTGCGGATCTGCCGACGGGTAGCGTGTACCCATTTTATGCGCTCAACGATGAACTGGCCGCGGCGCTGATGTATGATGAGCGTCACGCCACCCCAAATCCGGACACCCAGCATAAACTGGATGGCATCGATGCCGCGAAGTATGCGCGTCCAGCCGCGCTGACCATCTATTGCGATTATTTAAATATCAATTTTTGGAGTGCGCTGCAGAATAAAGGCGAGCTCTACCATCACCCACACGAAAGCGAACTGGTGGAGAGTATCCCGAGCGATATCTTTCCGGCCAGCGAAGCCGTGGTTCAGCAAGCTACCCAGTTTTTCAAAGATATTTTCAACACCCATTACGTGCCGCAGCCAACGCTGACCAGTGCGCGCATTTGGGAAGGCAACGTTAATTTTAACGTGCCGGAAAACCTCCAGTTCGGTTTTGGCGTGCACCAGTGGGCCATCGGCGCCAACGACAAAGAGGTGATCGAGGATCTGGTGGAACCGCTGCCGAATTTATTTACGTGCGGCGAAGCGTACAGCGACTACCAAGGTTGGGTTGAAGGCGCTTTACGCAGCACGGATCTCGTTCTGCAGAAAGGCTTCGGTCTGGCGCCACTGAGTGAAGTGTACGAGCAAGATCAAGGTCGCAGCAGCAGCGAAGCGATCCAAATCGCCTACCGCAAGATCAGCAACAAGATGATTATGGAATACATTGATCCGAACTTCAGCCCGAACACCAAACACAAGGTTACTTTAGCGGAAGTGAACAGCGTGCTCGGCGTGAATTTAAGCTACTTCGATAAACCG
