# Supplementary material for: Ancestral L-amino acid oxidases for deracemization and stereoinversion of amino acids
Source: Commun Chem. 2020 Dec 4;3:181. doi: 10.1038/s42004-020-00432-8 (PMC9814856; doi:10.1038/s42004-020-00432-8)
Supplement: Supplementary file 8 — Supplementary Data 5 [file 42004_2020_432_MOESM8_ESM.pdf]

wwPDB X-ray Structure Validation Summary Report ⓘ

CONFIDENTIAL VALIDATION REPORT

May 20, 2020 – 05:29 PM JST

PDB ID	:	7C4M
Title	:	Ancestral L-amino acid oxidase (AncLAAO-N5) L-Trp binding form
Deposited on	:	2020-05-18
Resolution	:	2.40 Å(reported)

This is a wwPDB X-ray Structure Validation Summary Report.

This report is produced by the wwPDB biocuration pipeline after annotation of the structure.

We welcome your comments at *validation@mail.wwpdb.org*
A user guide is available at
https://www.wwpdb.org/validation/2017/XrayValidationReportHelp
with specific help available everywhere you see the ⓘ symbol.

The following versions of software and data (see references ⓘ) were used in the production of this report:

MolProbity	:	4.02b-467
Mogul	:	1.8.5 (274361), CSD as541be (2020)
Xtriage (Phenix)	:	1.13
EDS	:	2.11
buster-report	:	1.1.7 (2018)
Percentile statistics	:	20171227.v01 (using entries in the PDB archive December 27th 2017)
Refmac	:	5.8.0158
CCP4	:	7.0.044 (Gargrove)
Ideal geometry (proteins)	:	Engh & Huber (2001)
Ideal geometry (DNA, RNA)	:	Parkinson et al. (1996)
Validation Pipeline (wwPDB-VP)	:	2.11

1 Overall quality at a glance (i)

The following experimental techniques were used to determine the structure:
X-RAY DIFFRACTION

The reported resolution of this entry is 2.40 Å.

Percentile scores (ranging between 0-100) for global validation metrics of the entry are shown in the following graphic. The table shows the number of entries on which the scores are based.

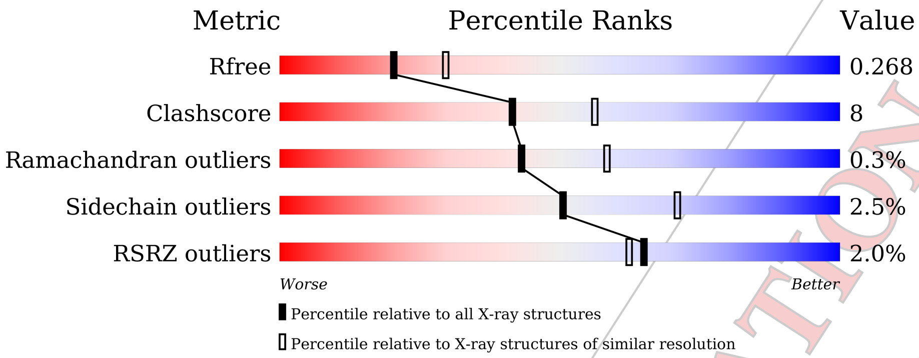

Metric	Whole archive (#Entries)	Similar resolution (#Entries, resolution range(Å))
R_{free}	111664	3481 (2.40-2.40)
Clashscore	122126	3956 (2.40-2.40)
Ramachandran outliers	120053	3897 (2.40-2.40)
Sidechain outliers	120020	3898 (2.40-2.40)
RSRZ outliers	108989	3386 (2.40-2.40)

The table below summarises the geometric issues observed across the polymeric chains and their fit to the electron density. The red, orange, yellow and green segments on the lower bar indicate the fraction of residues that contain outliers for >=3, 2, 1 and 0 types of geometric quality criteria respectively. A grey segment represents the fraction of residues that are not modelled. The numeric value for each fraction is indicated below the corresponding segment, with a dot representing fractions <=5% The upper red bar (where present) indicates the fraction of residues that have poor fit to the electron density. The numeric value is given above the bar.

Mol	Chain	Length	Quality of chain
1	A	663	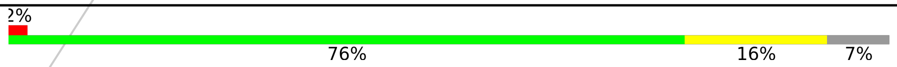

CONFIDENTIAL VALIDATION REPORT

2 Entry composition (i)

There are 4 unique types of molecules in this entry. The entry contains 5181 atoms, of which 0 are hydrogens and 0 are deuteriums.

In the tables below, the ZeroOcc column contains the number of atoms modelled with zero occupancy, the AltConf column contains the number of residues with at least one atom in alternate conformation and the Trace column contains the number of residues modelled with at most 2 atoms.

- Molecule 1 is a protein called Ancestral L-amino acid oxidase.

Mol	Chain	Residues	Atoms					ZeroOcc	AltConf	Trace
			Total	C	N	O	S			
1	A	614	4898	3134	813	935	16	0	0	0

- Molecule 2 is FLAVIN-ADENINE DINUCLEOTIDE (three-letter code: FAD) (formula: $C_{27}H_{33}N_9O_{15}P_2$) (labeled as "Ligand of Interest" by author).

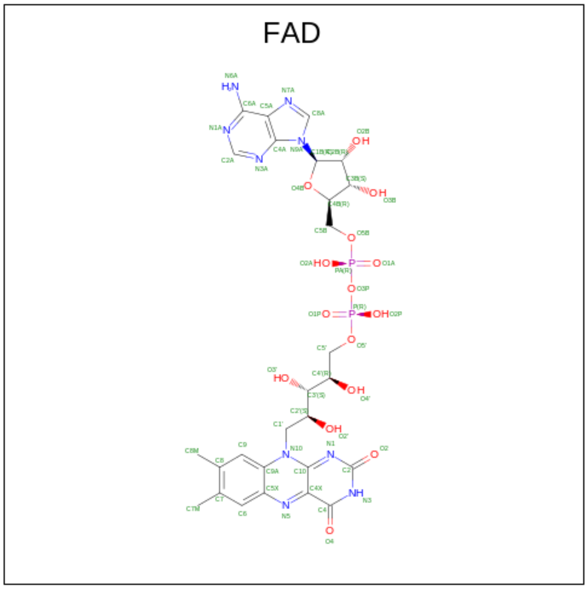

Mol	Chain	Residues	Atoms					ZeroOcc	AltConf
			Total	C	N	O	P		
2	A	1	53	27	9	15	2	0	0

- Molecule 3 is TRYPTOPHAN (three-letter code: TRP) (formula: $C_{11}H_{12}N_2O_2$).

CONFIDENTIAL - VALIDATION REPORT

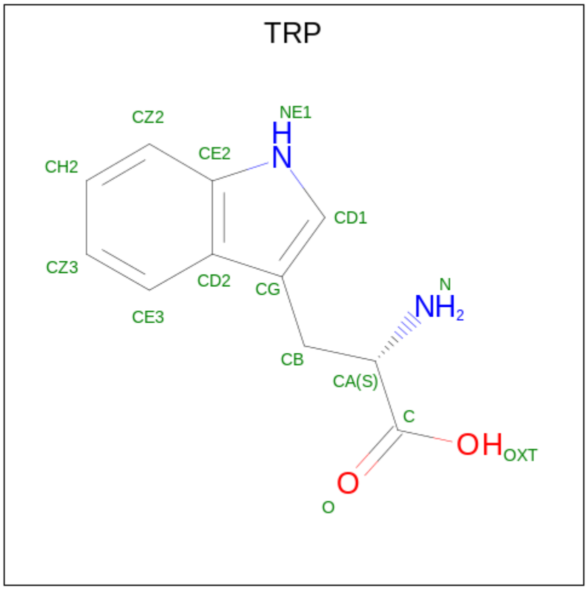

Mol	Chain	Residues	Atoms				ZeroOcc	AltConf
			Total	C	N	O		
3	A	1	15	11	2	2	0	0

- Molecule 4 is water.

Mol	Chain	Residues	Atoms		ZeroOcc	AltConf
			Total	O		
4	A	215	215	215	0	0

CONFIDENTIAL VALIDATION REPORT

3 Residue-property plots (i)

These plots are drawn for all protein, RNA and DNA chains in the entry. The first graphic for a chain summarises the proportions of the various outlier classes displayed in the second graphic. The second graphic shows the sequence view annotated by issues in geometry and electron density. Residues are color-coded according to the number of geometric quality criteria for which they contain at least one outlier: green = 0, yellow = 1, orange = 2 and red = 3 or more. A red dot above a residue indicates a poor fit to the electron density (RSRZ > 2). Stretches of 2 or more consecutive residues without any outlier are shown as a green connector. Residues present in the sample, but not in the model, are shown in grey.

- Molecule 1: Ancestral L-amino acid oxidase

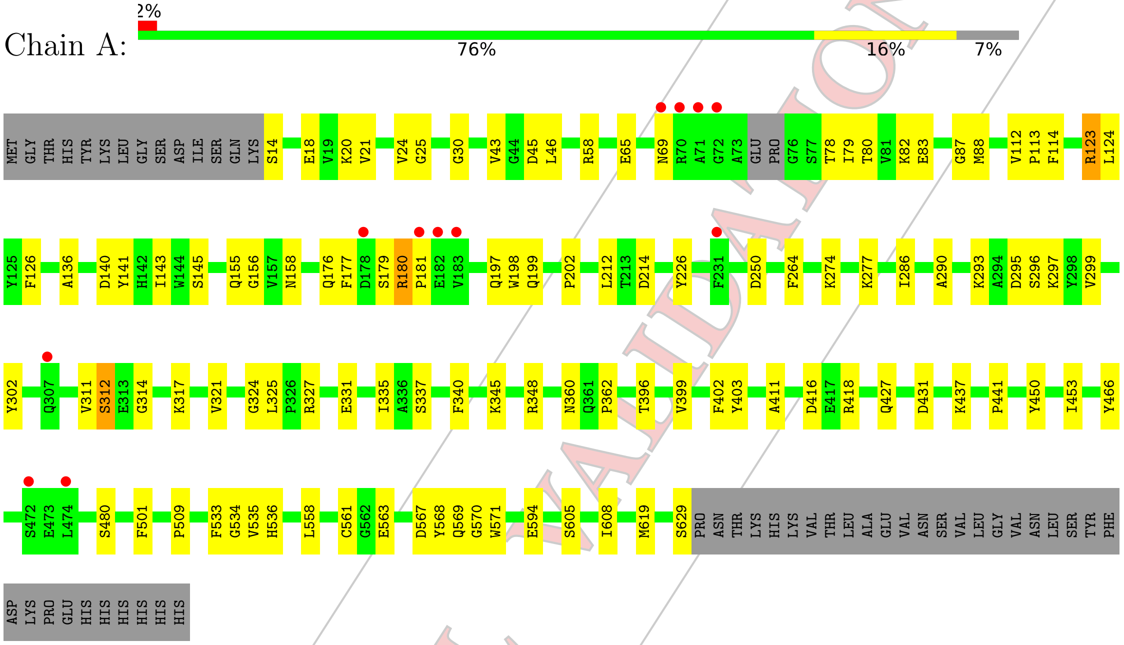

CONFIDENTIAL VALIDATION REPORT

4 Data and refinement statistics (i)

Property	Value	Source
Space group	I 4 2 2	Depositor
Cell constants<br>a, b, c, α, β, γ	132.50Å 132.50Å 191.73Å<br>90.00° 90.00° 90.00°	Depositor
Resolution (Å)	47.93 – 2.40<br>47.93 – 2.40	Depositor<br>EDS
% Data completeness<br>(in resolution range)	100.0 (47.93-2.40)<br>100.0 (47.93-2.40)	Depositor<br>EDS
R_{merge}	0.11	Depositor
R_{sym}	(Not available)	Depositor
$< I/\sigma(I) >$ [1]	4.10 (at 2.39Å)	Xtriage
Refinement program	PHENIX 1.14_3260	Depositor
R, R_{free}	0.208 , 0.270<br>0.208 , 0.268	Depositor<br>DCC
R_{free} test set	1617 reflections (4.82%)	wwPDB-VP
Wilson B-factor (Å^2)	35.5	Xtriage
Anisotropy	0.003	Xtriage
Bulk solvent k_{sol}(e/Å^3), B_{sol}(Å^2)	0.33 , 45.5	EDS
L-test for twinning[2]	$< \|L\| > = 0.42$, $< L^2 > = 0.24$	Xtriage
Estimated twinning fraction	0.066 for -1/2*h-1/2*k-1/2*l,-1/2*h-1/2*k+1/2*l,-h+k<br>0.045 for -1/2*h+1/2*k-1/2*l,1/2*h-1/2*k-1/2*l,-h-k	Xtriage
F_o,F_c correlation	0.92	EDS
Total number of atoms	5181	wwPDB-VP
Average B, all atoms (Å^2)	37.0	wwPDB-VP

Xtriage's analysis on translational NCS is as follows: *The largest off-origin peak in the Patterson function is 2.75% of the height of the origin peak. No significant pseudotranslation is detected.*

[1]Intensities estimated from amplitudes.

[2]Theoretical values of $< |L| >$, $< L^2 >$ for acentric reflections are 0.5, 0.333 respectively for untwinned datasets, and 0.375, 0.2 for perfectly twinned datasets.

CONFIDENTIAL VALIDATION REPORT

5 Model quality (i)

5.1 Standard geometry (i)

Bond lengths and bond angles in the following residue types are not validated in this section: FAD

The Z score for a bond length (or angle) is the number of standard deviations the observed value is removed from the expected value. A bond length (or angle) with $|Z| > 5$ is considered an outlier worth inspection. RMSZ is the root-mean-square of all Z scores of the bond lengths (or angles).

Mol	Chain	Bond lengths		Bond angles	
		RMSZ	$\#|Z|>5$	RMSZ	$\#|Z|>5$
1	A	0.42	0/5023	0.56	0/6818

There are no bond length outliers.

There are no bond angle outliers.

There are no chirality outliers.

There are no planarity outliers.

5.2 Too-close contacts (i)

In the following table, the Non-H and H(model) columns list the number of non-hydrogen atoms and hydrogen atoms in the chain respectively. The H(added) column lists the number of hydrogen atoms added and optimized by MolProbity. The Clashes column lists the number of clashes within the asymmetric unit, whereas Symm-Clashes lists symmetry related clashes.

Mol	Chain	Non-H	H(model)	H(added)	Clashes	Symm-Clashes
1	A	4898	0	4722	74	0
2	A	53	0	31	5	0
3	A	15	0	9	1	0
4	A	215	0	0	8	1
All	All	5181	0	4762	74	1

The all-atom clashscore is defined as the number of clashes found per 1000 atoms (including hydrogen atoms). The all-atom clashscore for this structure is 8.

The worst 5 of 74 close contacts within the same asymmetric unit are listed below, sorted by their clash magnitude.

CONFIDENTIAL VALIDATION REPORT

Atom-1	Atom-2	Interatomic distance (Å)	Clash overlap (Å)
1:A:88:MET:CE	1:A:402:PHE:HZ	1.79	0.94
1:A:87:GLY:HA2	2:A:701:FAD:N5	1.97	0.80
1:A:88:MET:CE	1:A:402:PHE:CZ	2.68	0.77
1:A:180:ARG:H	1:A:181:PRO:HD2	1.51	0.73
1:A:535:VAL:HG11	3:A:702:TRP:CZ2	2.23	0.73

All (1) symmetry-related close contacts are listed below. The label for Atom-2 includes the symmetry operator and encoded unit-cell translations to be applied.

Atom-1	Atom-2	Interatomic distance (Å)	Clash overlap (Å)
4:A:945:HOH:O	4:A:947:HOH:O[16_554]	2.16	0.04

5.3 Torsion angles (i)

5.3.1 Protein backbone (i)

In the following table, the Percentiles column shows the percent Ramachandran outliers of the chain as a percentile score with respect to all X-ray entries followed by that with respect to entries of similar resolution.

The Analysed column shows the number of residues for which the backbone conformation was analysed, and the total number of residues.

Mol	Chain	Analysed	Favoured	Allowed	Outliers	Percentiles	
1	A	610/663 (92%)	580 (95%)	28 (5%)	2 (0%)	43	58

All (2) Ramachandran outliers are listed below:

Mol	Chain	Res	Type
1	A	180	ARG
1	A	450	TYR

5.3.2 Protein sidechains (i)

In the following table, the Percentiles column shows the percent sidechain outliers of the chain as a percentile score with respect to all X-ray entries followed by that with respect to entries of similar resolution.

The Analysed column shows the number of residues for which the sidechain conformation was analysed, and the total number of residues.

CONFIDENTIAL VALIDATION REPORT

Mol	Chain	Analysed	Rotameric	Outliers	Percentiles	
1	A	525/570 (92%)	512 (98%)	13 (2%)	50	71

5 of 13 residues with a non-rotameric sidechain are listed below:

Mol	Chain	Res	Type
1	A	250	ASP
1	A	264	PHE
1	A	480	SER
1	A	226	TYR
1	A	312	SER

Some sidechains can be flipped to improve hydrogen bonding and reduce clashes. All (1) such sidechains are listed below:

Mol	Chain	Res	Type
1	A	199	GLN

5.3.3 RNA (i)

There are no RNA molecules in this entry.

5.4 Non-standard residues in protein, DNA, RNA chains (i)

There are no non-standard protein/DNA/RNA residues in this entry.

5.5 Carbohydrates (i)

There are no carbohydrates in this entry.

5.6 Ligand geometry (i)

2 ligands are modelled in this entry.

In the following table, the Counts columns list the number of bonds (or angles) for which Mogul statistics could be retrieved, the number of bonds (or angles) that are observed in the model and the number of bonds (or angles) that are defined in the Chemical Component Dictionary. The Link column lists molecule types, if any, to which the group is linked. The Z score for a bond length (or angle) is the number of standard deviations the observed value is removed from the expected value. A bond length (or angle) with $|Z| > 2$ is considered an outlier worth inspection. RMSZ is the root-mean-square of all Z scores of the bond lengths (or angles).

CONFIDENTIAL VALIDATION REPORT

Mol	Type	Chain	Res	Link	Bond lengths			Bond angles		
					Counts	RMSZ	#|Z| > 2	Counts	RMSZ	#|Z| > 2
2	FAD	A	701	-	51,58,58	1.25	5 (9%)	60,89,89	2.21	7 (11%)

In the following table, the Chirals column lists the number of chiral outliers, the number of chiral centers analysed, the number of these observed in the model and the number defined in the Chemical Component Dictionary. Similar counts are reported in the Torsion and Rings columns. '-' means no outliers of that kind were identified.

Mol	Type	Chain	Res	Link	Chirals	Torsions	Rings
2	FAD	A	701	-	-	9/30/50/50	0/6/6/6

All (5) bond length outliers are listed below:

Mol	Chain	Res	Type	Atoms	Z	Observed(Å)	Ideal(Å)
2	A	701	FAD	C4X-C10	5.56	1.44	1.38
2	A	701	FAD	C4-N3	3.26	1.38	1.33
2	A	701	FAD	C4X-N5	-2.99	1.29	1.33
2	A	701	FAD	C4-C4X	2.35	1.45	1.41
2	A	701	FAD	C9A-N10	2.29	1.41	1.38

The worst 5 of 7 bond angle outliers are listed below:

Mol	Chain	Res	Type	Atoms	Z	Observed(^{o})	Ideal(^{o})
2	A	701	FAD	C4-N3-C2	12.49	125.69	115.14
2	A	701	FAD	C4X-C4-N3	-6.95	113.92	123.43
2	A	701	FAD	C10-C4X-N5	4.96	124.69	121.26
2	A	701	FAD	C4-C4X-C10	-3.54	117.61	119.95
2	A	701	FAD	C4X-C10-N10	-3.44	116.77	120.30

There are no chirality outliers.

5 of 9 torsion outliers are listed below:

Mol	Chain	Res	Type	Atoms
2	A	701	FAD	C2'-C3'-C4'-C5'
2	A	701	FAD	PA-O3P-P-O5'
2	A	701	FAD	O3'-C3'-C4'-O4'
2	A	701	FAD	C2'-C3'-C4'-O4'
2	A	701	FAD	O4B-C4B-C5B-O5B

There are no ring outliers.

1 monomer is involved in 5 short contacts:

CONFIDENTIAL VALIDATION REPORT

Mol	Chain	Res	Type	Clashes	Symm-Clashes
2	A	701	FAD	5	0

The following is a two-dimensional graphical depiction of Mogul quality analysis of bond lengths, bond angles, torsion angles, and ring geometry for all instances of the Ligand of Interest. In addition, ligands with molecular weight > 250 and outliers as shown on the validation Tables will also be included. For torsion angles, if less then 5% of the Mogul distribution of torsion angles is within 10 degrees of the torsion angle in question, then that torsion angle is considered an outlier. Any bond that is central to one or more torsion angles identified as an outlier by Mogul will be highlighted in the graph. For rings, the root-mean-square deviation (RMSD) between the ring in question and similar rings identified by Mogul is calculated over all ring torsion angles. If the average RMSD is greater than 60 degrees and the minimal RMSD between the ring in question and any Mogul-identified rings is also greater than 60 degrees, then that ring is considered an outlier. The outliers are highlighted in purple. The color gray indicates Mogul did not find sufficient equivalents in the CSD to analyse the geometry.

Ligand FAD A 701

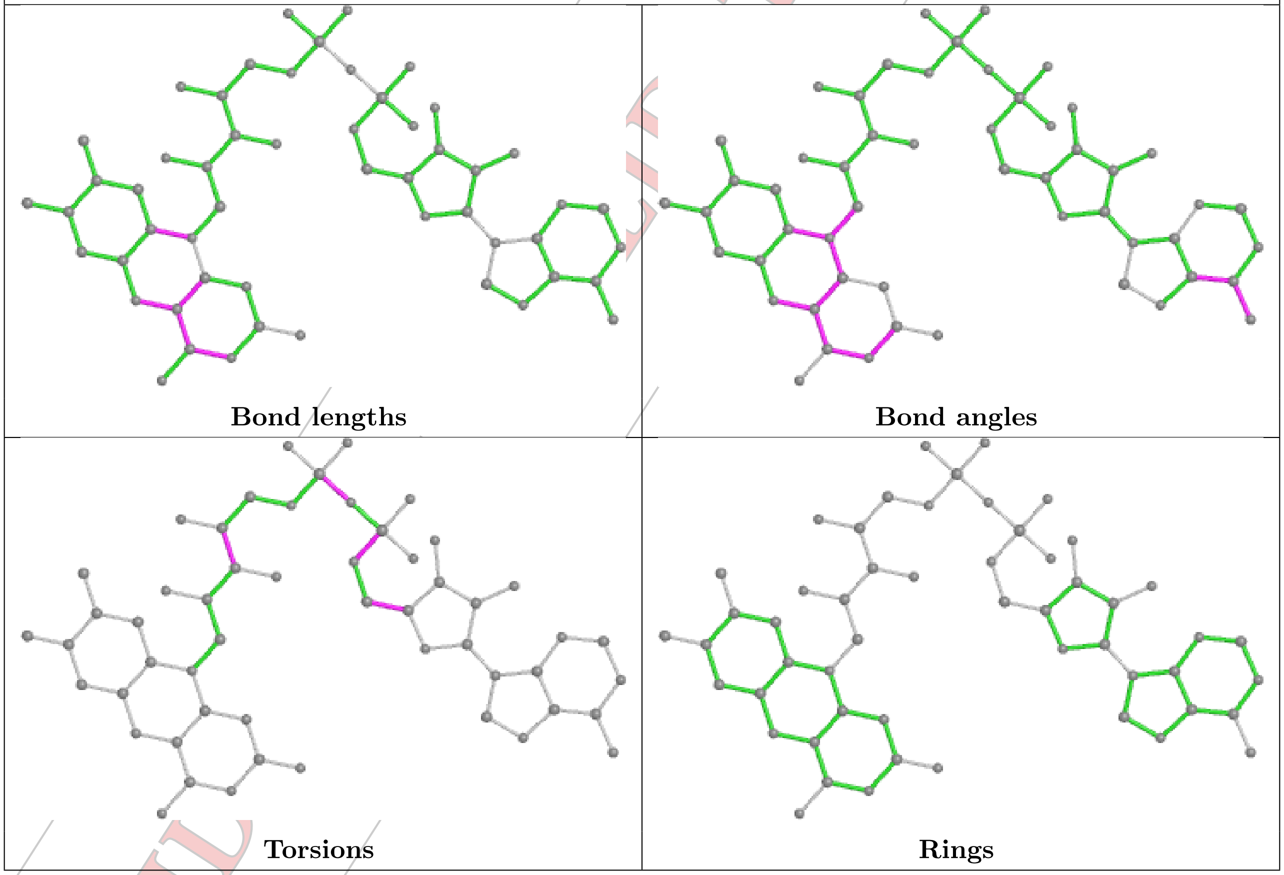

5.7 Other polymers (i)

There are no such residues in this entry.

5.8 Polymer linkage issues (i)

There are no chain breaks in this entry.

CONFIDENTIAL VALIDATION REPORT

6 Fit of model and data (i)

6.1 Protein, DNA and RNA chains (i)

In the following table, the column labelled '#RSRZ> 2' contains the number (and percentage) of RSRZ outliers, followed by percent RSRZ outliers for the chain as percentile scores relative to all X-ray entries and entries of similar resolution. The OWAB column contains the minimum, median, 95th percentile and maximum values of the occupancy-weighted average B-factor per residue. The column labelled 'Q< 0.9' lists the number of (and percentage) of residues with an average occupancy less than 0.9.

Mol	Chain	Analysed	<RSRZ>	#RSRZ>2			OWAB(Å^2)	Q<0.9
1	A	614/663 (92%)	-0.12	12 (1%)	65	62	24, 35, 59, 80	0

The worst 5 of 12 RSRZ outliers are listed below:

Mol	Chain	Res	Type	RSRZ
1	A	71	ALA	6.4
1	A	474	LEU	3.5
1	A	72	GLY	3.0
1	A	69	ASN	2.9
1	A	182	GLU	2.9

6.2 Non-standard residues in protein, DNA, RNA chains (i)

There are no non-standard protein/DNA/RNA residues in this entry.

6.3 Carbohydrates (i)

There are no carbohydrates in this entry.

6.4 Ligands (i)

In the following table, the Atoms column lists the number of modelled atoms in the group and the number defined in the chemical component dictionary. The B-factors column lists the minimum, median, 95th percentile and maximum values of B factors of atoms in the group. The column labelled 'Q< 0.9' lists the number of atoms with occupancy less than 0.9.

Mol	Type	Chain	Res	Atoms	RSCC	RSR	B-factors(Å^2)	Q<0.9
3	TRP	A	702	15/15	0.86	0.25	38,47,52,54	0
2	FAD	A	701	53/53	0.95	0.14	26,33,39,40	0

CONFIDENTIAL VALIDATION REPORT

The following is a graphical depiction of the model fit to experimental electron density of all instances of the Ligand of Interest. In addition, ligands with molecular weight > 250 and outliers as shown on the geometry validation Tables will also be included. Each fit is shown from different orientation to approximate a three-dimensional view.

Electron density around FAD A 701:

2mF$_o$-DF$_c$ (at 0.7 rmsd) in gray
mF$_o$-DF$_c$ (at 3 rmsd) in purple (negative) and green (positive)

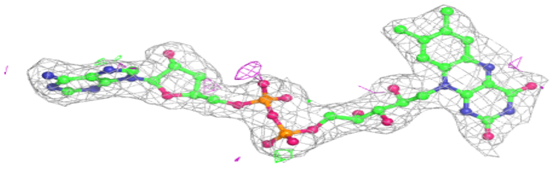

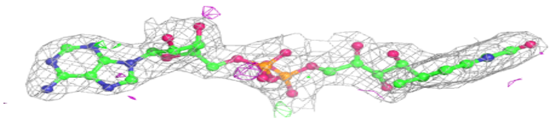

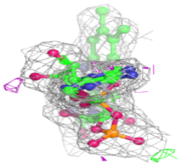

6.5 Other polymers (i)

There are no such residues in this entry.

CONFIDENTIAL VALIDATION REPORT
